# Advanced deep learning techniques for automated license plate recognition

**DOI:** 10.1038/s41598-025-24967-9

**Published:** 2025-11-21

**Authors:** Phayung Meesad, Wichan Thumthong

**Affiliations:** 1 Department of Information Technology Management, Faculty of Information Technology and Digital Innovation, King Mongkut’s University of Technology North Bangkok (KMUTNB), Pracharat 1 Rd., Bangsue, Bangkok, 10800 Thailand; 2 Department of Information Technology, Faculty of Information Technology and Digital Innovation, King Mongkut’s University of Technology North Bangkok (KMUTNB), Pracharat 1 Rd., Bangsue, Bangkok, 10800 Thailand

**Keywords:** Automated license plate recognition, Deep learning, YOLO, Tesseract OCR, Thai license plate recognition, Object detection, Image processing, Electrical and electronic engineering, Computational science, Computer science, Information technology

## Abstract

This research improves the capacity of automated license plate recognition (ALPR) to meet not only the needs of its methodology but also those it is confronted with in everyday situations. By combining YOLOv10 with a specially customized Tesseract OCR engine, the aim was to achieve the recognition of the Thai–Roman mixed-script car license plates, which represents a difficult and scarcely resolved problem in the literature. To ensure the system can be thoroughly tested in a wide range of scenarios, we have assembled a large-scale dataset that comprises 50,000 images and 10,000 video clips depicting different lighting and weather conditions. We have tested real-time capability on Jetson Nano and our results support the possibility of scaling for intelligent transportation systems. Comparing our experiments to the results of the latest detectors (YOLOv5, YOLOv8, YOLOv9, Faster R-CNN, SSD), we find that YOLOv10 consistently gives better results with detection accuracy of 99.16%, an F1-score of 0.992, and an inference time of 1.0 ms/image, while under severe conditions there is no significant decrease of performance. In sum, these empirical results turn the proposed system into a both novel and practical contribution for regionally adaptive ALPR research.

## Introduction

Automated License Plate Recognition (ALPR) systems have become essential for intelligent transportation and security infrastructures. ANPR systems can use video streams and images to capture the license plate data, which is fundamental for managing traffic, protecting public safety, collecting tolls, and providing secure facility access. The increasing development of smart cities has amplified the demand for reliable and adaptive ALPR systems, necessitating advanced technologies to address region-specific and environmental challenges^1^.

However, current ALPR systems encounter significant challenges when applied to Thai license plates, which feature mixed Thai and Roman scripts, a wide range of font styles, and diverse color schemes across private, commercial, and government vehicles. These region-specific characteristics complicate detection and recognition processes, often leading to reduced accuracy and inconsistent system performance in real-world environments^2,3^. Although most ALPR systems utilize image processing methods, such as edge detection and morphological operations, these systems can work effectively only when there are no changes in lighting and weather conditions. If changes in lighting and weather conditions occur in the environment, the systems’ accuracy will be unstable. To solve these challenges effectively, we should leverage modern, diverse technologies—especially those tailored to local needs^4^.

Recent advancements in deep learning, particularly through Convolutional Neural Networks (CNNs), have significantly improved the accuracy and reliability of Automatic License Plate Recognition (ALPR) systems. Models such as YOLO (You Only Look Once)^5^ have demonstrated exceptional real-time object detection capabilities, with successive versions introducing critical improvements. YOLOv3 incorporated multi-scale predictions to detect small objects better, while YOLOv4^6^ introduced Cross Stage Partial (CSP) connections to improve efficiency and accuracy. However, despite these advancements, applying such models to complex, region-specific license plates, such as those in Thailand, remains challenging^2,7^.

Optical Character Recognition (OCR) plays a critical role in ALPR systems by extracting alphanumeric information from detected license plates. Tesseract OCR, renowned for its robustness and adaptability to various languages and font styles, was fine-tuned in this study to accommodate Thai license plates’ dual-script nature and typographic variability. While Tesseract offered high recognition accuracy and seamless integration into post-processing pipelines, alternative lightweight OCR engines like EasyOCR and PaddleOCR were explored but showed notable limitations in handling complex layouts, stylized fonts, and script switching — particularly under real-world conditions involving Thai characters.

While Tesseract OCR demonstrated reliable performance in recognizing Thai license plate characters^8^, alternative engines such as EasyOCR and PaddleOCR exhibit limitations in handling complex fonts and scripts. Future research will systematically benchmark these OCR technologies to enhance system flexibility and accuracy further and assess their potential for integration within the proposed ALPR framework. Identifying an optimal OCR solution is crucial for developing a scalable and robust ALPR application adaptable to diverse regional requirements.

Thai vehicle license plates present a more complex recognition challenge due to their use of Thai and Roman scripts. Most conventional ALPR systems are not designed to accurately detect or interpret Thai characters, leading to suboptimal performance in this context^2^. Furthermore, multiple font styles on Thai plates add a layer of variability, making character recognition significantly more difficult^9^.

Thai license plates vary significantly in design based on the type of vehicle, including private, commercial, and government vehicles. The variation might be in the color schemes and the designs, as the old-fashioned ALPR systems, which depended on unchanging design features for identifying and reading plates, are no longer functional. A direct result of this is that the recognitions are either futile or incorrect. The unwavering operation of legacy ALPR technology can be compromised by environmental conditions, such as variations in lighting levels and likely weather changes outside. Their frequently intricate designs make Thai plates complicated to read under poor lighting or adverse weather conditions. Systems may fail to accurately capture and recognize plates in low-light situations or when plates are partially obscured by dirt or rain, leading to inconsistent performance readings^2,10^. Many current ALPR systems struggle with real-time processing demands, particularly when faced with the additional complexity of Thai license plates. Traditional systems can easily become overwhelmed by the need to process numerous plate designs and scripts accurately and quickly in real-time, leading to lag and a decrease in accuracy levels in places with high visitor traffic^11^.

In this research, a novel end-to-end recognition framework is presented as a solution to the problems with the current ALPR systems, especially those of the Thai license plate, and it was named the enhanced one. In this work, YOLOv10 object detection and a fine-tuned version of Tesseract OCR have been used to create a combined system that can meet the challenges of dual-script encoding, varying font styles and real-world environmental variability with the highest precision.

This paper mainly contributes to the field in the following four key contributions:

1. Methodological innovation: We present a system that uses YOLOv10 together with a specially trained Tesseract OCR engine for which the preprocessing (denoising, contrast normalization, and segmentation) has been carried out. With this a robust identification of the Thai–Roman mixed script on license plates is possible, which in turn is a challenge that has been solved only to a very limited extent in previous ALPR research.
2. Dataset contribution: We create a dataset with 50,000 photos and 10,000 videos (10–15 seconds each) of Thai automobiles recorded under various scenarios (day, night, rain, fog). This dataset is one of the most realistic benchmarks for evaluating ALPR systems in non-standardized contexts and therefore provides a very high amount of realism.
3. Comparative benchmarking: We conduct extensive experiments to compare the performance of YOLOv10 with that of five other state-of-the-art detectors (YOLOv5, YOLOv8, YOLOv9, Faster R-CNN, SSD). YOLOv10 gives the best results for all the metrics—99.16% accuracy, 0.992 F1-score, and 1.0 ms/image inference time—plus keeps its performance even when there are some bad conditions.
4. Real-world deployment: We test the system on NVIDIA Jetson Nano and confirm that it can run up to 30 FPS in real time. So, the ALPR proposal is not only accurate in lab experiments but also at least as good as when it comes to intelligent transport and smart mobility applications.

When these contributions work together, they not only pioneer the system as a new solution for ALPR but also move beyond simply creating a dataset for regionally adaptive license plate recognition.

To make it easier to follow, a complete list of the shortened terms that were referred to throughout the research work is given in Supplementary Table 1.

## Literature review

Traditional Automated License Plate Recognition (ALPR) systems typically involve three stages: license plate localization, character segmentation, and character recognition. Early approaches relied on image processing techniques such as thresholding, edge detection, and morphological operations to isolate and interpret license plate regions. However, these methods exhibited limited robustness, particularly under challenging conditions such as variable lighting, occlusion, and diverse plate designs, motivating the transition toward deep learning-based solutions^12^.

Despite notable progress in Automatic License Plate Recognition (ALPR) through deep learning and Optical Character Recognition (OCR) technologies, existing research often falls short in addressing region-specific complexities and environmental variability. Traditional image processing methods struggle to maintain consistent performance under fluctuating lighting and weather conditions. Even state-of-the-art deep learning models, while effective in controlled settings, remain under-optimized for handling the intricacies of non-standard license plates, such as those found in Thailand^2,13^.

In regions such as the United States and Europe, where plate formats are standardized, deep learning models have significantly improved detection and recognition accuracy^12,14^. However, studies explicitly designed for Thai license plates are comparatively scarce, indicating a critical gap in the literature^2,9,13^. The challenge of recognizing Thai plates stems from several unique characteristics, including the use of both Roman and Thai scripts, a wide variety of fonts, and the presence of color-coded backgrounds that denote different vehicle types^7,10^.

The evolution of the YOLO (You Only Look Once) family of object detection models has substantially improved real-time ALPR capabilities. YOLOv3 introduced multi-scale prediction to enhance small object detection performance, particularly beneficial for fine-grained license plate features^15^. YOLOv4 improved speed and accuracy by incorporating Cross Stage Partial (CSP) connections, resulting in better feature reuse and reduced computation^6^. YOLOv5, developed by Ultralytics, introduced a streamlined architecture with improved mean Average Precision (mAP), faster inference, and ease of deployment, making it suitable for real-time embedded applications^16,17^. Subsequent iterations, including YOLOv6 and YOLOv7, leveraged architectural enhancements such as Spatial Pyramid Pooling (SPP) and Path Aggregation Networks (PAN) to further boost detection precision and speed, with YOLOv7 demonstrating state-of-the-art real-time accuracy through advanced training strategies^18^. The latest versions, YOLOv8 to YOLOv10, further refined these innovations by introducing Dynamic Convolutional Kernels (DCK) and Advanced Augmentation Strategies (AAS), enabling greater robustness across varied conditions — a critical factor for recognizing non-standard or dual-script plates in regions such as Thailand^19,20^. These advancements, combined with the general progression of Convolutional Neural Networks (CNNs) in object detection and image classification tasks^21^, form the backbone of modern, high-performance ALPR systems.

Optical Character Recognition (OCR) engines are critical for extracting alphanumeric information in ALPR systems, particularly when handling non-standard fonts, regional scripts, and adverse imaging conditions. Tesseract OCR, an open-source and highly adaptable engine maintained by Google, has been widely adopted due to its robust multilingual support and flexibility in customization^22^. However, alternative OCR engines, such as EasyOCR, have shown limitations in recognizing complex character sets and stylized formats, especially in dual-script environments like Thai license plates^11,23^. Addressing these challenges requires coupling robust OCR technology with high-accuracy object detectors. Integrating YOLO-based models — particularly recent versions such as YOLOv4 and YOLOv10 — with fine-tuned Tesseract enhances end-to-end ALPR performance by ensuring precise detection and reliable character recognition under real-world conditions^6^. For instance, Puarungroj and Boonsirisumpun demonstrated that tailoring Tesseract to the Thai script substantially improves recognition rates on local license plates^2^, while Poorani et al. successfully implemented a YOLOv4 + Tesseract pipeline for accurate detection and recognition on custom datasets^24^.

While deep learning models and OCR technologies have significantly advanced ALPR systems, several limitations persist. Many existing studies prioritize standardized license plate formats and neglect region-specific complexities, such as the dual-script, stylized, or variably formatted Thai plates. Moreover, the integration of object detection frameworks and OCR engines has yet to be comprehensively optimized for these nuanced recognition challenges. As noted by Fasha et al.^25^, hybrid models that blend the strengths of multiple architectures offer a promising path forward, particularly when combined with hardware-aware optimization strategies. Furthermore, enhanced data augmentation techniques are necessary to ensure robustness across various lighting conditions, obstructions, and motion blur. Another important consideration is the limited capacity of widely used OCR engines, such as EasyOCR, in handling regionally complex scripts, as documented in^23^. Future research must address these challenges to develop scalable and adaptable ALPR systems that can maintain high performance in diverse real-world deployments, particularly within intelligent transportation systems in Southeast Asia.

A comparative study of the most recent research on ALPR focusing on Thai or regionally complex plates is tabulated in Table 1. The table highlights the models and techniques that were used, the main advantages and disadvantages as well as the performance metrics presented by each research. It exposes the fact that although different methods like Single Shot Multibox Detector (SSD)-based detectors and YOLO variants performed well under conventional settings, their adaptation to Thai license plates—containing dual scripts, variable fonts, and environmental constraints of various kinds—has not been uniformly successful. Also, OCR implementation, especially for Thai characters, is still an area that has been largely overlooked in most frameworks. This explanation not only points out the kinds of things that have been ignored in the various License Plate Recognition systems, but it also provides evidence for the proposal of a more powerful and geographically-constrained system in this research.

**Table 1:** Comparative summary of recent ALPR studies on Thai license plates, highlighting models used, core advantages, limitations, and reported performance metrics.

Paper/Authors (Year)	Models/techniques used	Advantages	Disadvantages	Performance parameters
Puarungroj & Boonsirisumpun^2^	MobileNet, Inception-v3	Focus on Thai motorcycle plates Simple system integration	Limited dataset Misclassification of similar Thai characters	91.76% accuracy using MobileNet on 120 images (886 characters)
Sainui et al.^11^	SSD MobileNetV2 + EasyOCR	Works on video input Post-processing for plate filtering Better Thai character recognition than Tesseract	Blurred images reduce accuracy Requires stopping at checkpoints Resolution limitations	Image: 99% detection, 72% plate recog., 92% character recog. Video: 91.70% detection, 83.46% plate recog., 96.99% character recog.
Kitvimonrat & Watcharabutsarakham^13^	SSD + MobileNet, EAST, Bi-LSTM + CTC	Handles distortion and rotation Robust to viewing angles	Limited metric details Complex implementation	“High accuracy rate” for distorted plates (exact metrics not provided)
Poorani et al.^24^	YOLOv4 + Tesseract OCR	Versatile across environments Efficient preprocessing Low computational cost	OCR struggles without fine-tuning Limited to specific viewing angles	Detection: 92% accuracy OCR: 81% accuracy
Kraisin^9^	YOLO + traditional ML for components	Handles low-quality images Real-world effectiveness Component-based recognition	Multi-stage system Separate models for different characters	86.45% overall accuracy on full plate recognition
Wang et al.^26^	Deep networks with defogging and enhancement	Robust to adverse conditions Uses super-resolution and defogging Two systems: W-LPR, SC-LPR	Developed for Korean plates Needs adaptation for Thai	98.94% recognition accuracy
Tesseract vs. Google Cloud Vision^27^	Tesseract OCR vs. Google Vision API	Comparative Thai document study Evaluated preprocessing effects	Focused on registration documents Not designed for license plates	Google Vision: 84.43% accuracy Tesseract: 47.02% on Thai documents

To sum up, the studies done in the field of automatic license plate recognition (ALPR) in Thailand and similar areas have been successful to some extent, but they have also pointed out some limitations. The research work that used YOLOv3 and YOLOv4 led to fairly good accuracy; however, the systems they built were not very robust, environmental variations such as low light, and bad weather would make it difficult for them to function correctly. The majority of initial works were limited by the small size of the datasets (usually less than 10,000 samples), which means the results presented might not be valid for other datasets. Besides that, the problem of Thai–Roman mixed-script plates, a highly prevalent issue in practice, has barely been addressed in the literature. Furthermore, most of the prior methods were usually tested only in controlled environments, without clear indications of how they would perform when implemented on resource-limited edge devices. These shortcomings in the field have been the reasons for the author to use YOLOv10 combined with a properly trained OCR engine, to take the advantage of a large-scale dataset (50,000 images and 10,000 video clips), and to do the validation of the real-time performance on Jetson Nano.

### Identified research gap and proposed contributions

Despite notable advancements in ALPR systems driven by deep learning and OCR technologies, many existing studies overlook region-specific complexities and fail to address diverse environmental conditions. Traditional image processing approaches often lack robustness in variable lighting conditions, weather, or motion-blurred scenarios, limiting their applicability in real-world deployments. Moreover, even state-of-the-art deep learning models have not been comprehensively optimized to accommodate Thai license plates’ structural and script-related intricacies, often including dual-script combinations, stylized fonts, and non-standard layouts. These limitations underscore the need for regionally adaptive ALPR frameworks integrating advanced detection architectures with customized OCR solutions for robust character recognition in challenging environments.

This research addresses these gaps by developing a specialized ALPR system tailored to Thai license plates. By integrating state-of-the-art YOLO object detection models with fine-tuned Tesseract OCR, the study proposes a robust and scalable solution that meets the complex requirements of Thai license plate recognition. The approach combines a diverse dataset, effective preprocessing techniques, and the latest innovations in model architecture to improve the accuracy and resilience of ALPR systems under real-world conditions.

The proposed method contributes to the field of Automated License Plate Recognition (ALPR) in several key areas:

*Integration of cutting-edge technologies*: The system combines YOLOv10 with a fine-tuned Tesseract OCR engine, achieving high accuracy and speed in recognizing complex Thai license plates. This integration addresses limitations in previous ALPR systems, specifically regarding detection reliability and script adaptability.

*Region-specific optimization*: Unlike conventional ALPR models focusing on standardized Western plates, this method is explicitly designed for Thai license plates. It handles hybrid scripts (Thai and Roman), varying font styles, and category-specific formats across private, commercial, and governmental vehicles.

*Robustness across environmental conditions*: Through extensive data acquisition and preprocessing, the system demonstrates consistent performance under fog, rain, low light, and motion-induced blur conditions common in real deployments.

*Comprehensive dataset and preprocessing*: A dataset comprising 50,000 annotated images and 10,000 video segments supports model generalizability. Advanced preprocessing techniques enhance training robustness, including noise filtering, augmentation, and normalization.

*Real-time processing and scalability*: Achieving inference speeds of 1.0 ms per image with YOLOv10, the system is suitable for real-time applications like smart traffic monitoring and toll collection. Successful deployment on edge hardware (e.g., NVIDIA Jetson Nano) demonstrates its scalability to low-resource environments.

*Advanced methodological contributions*: The inclusion of Dynamic Convolutional Kernels (DCK) and Advanced Augmentation Strategies (AAS) in the YOLOv10 backbone strengthens the model’s robustness and adaptability, contributing novel enhancements to object detection research.

*Practical applications and insights*: The proposed framework provides operational guidance for ALPR deployment in smart cities and intelligent transportation systems, bridging the gap between academic innovation and real-world impact.

*Benchmarking and comparative analysis*: The model is benchmarked against Faster Region-based Convolutional Neural Network (R-CNN), SSD, and earlier YOLO versions. This comparison informs trade-off decisions and future optimization strategies.

*Open research opportunities*: The study outlines directions for future work, including cross-regional transfer learning, multimodal data integration (e.g., audio and video), and enhancing edge deployment for large-scale adoption.

This methodology bridges the gap between region-specific requirements and global research trends, offering a robust, scalable, and high-performing ALPR solution for intelligent urban mobility and transportation systems.

**Fig. 1:**
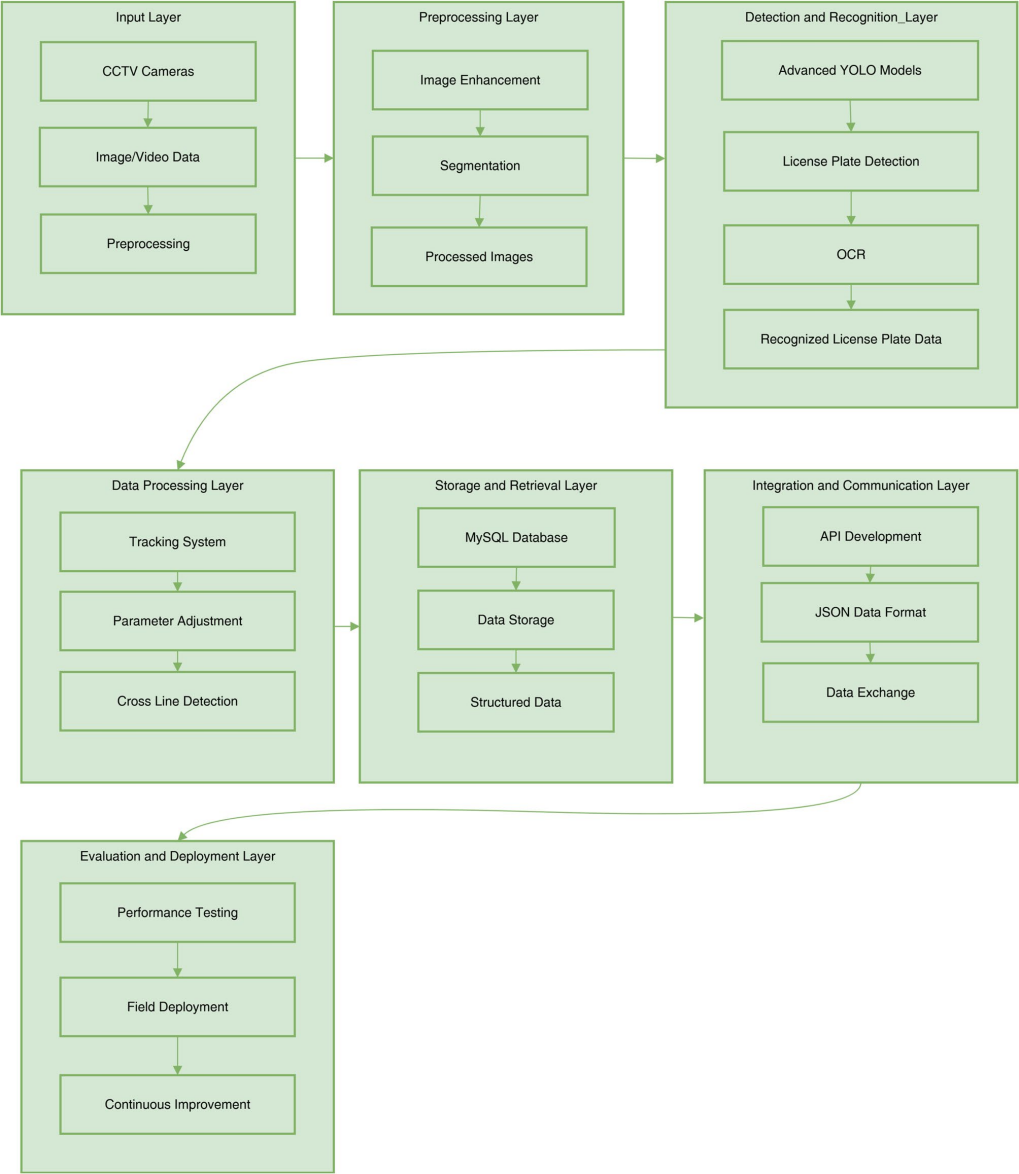
The Proposed Automatic License Plate Recognition (ALPR).

## Methodology

This section presents the proposed ALPR system architecture to achieve high-accuracy vehicle license plate detection and recognition under diverse real-world conditions. The system’s architecture is composed of seven modular parts, starting from data acquisition to integration. As a unit, they confirm that the ALPR solution is durable, flexible, and able to perform in real-time. Figure 1 illustrates the overall architecture of the system.

The system’s input layer captures real-time image and video streams, primarily sourced from Closed-Circuit Television (CCTV) cameras and vehicular dashcams. After acquisition, the preprocessing layer enhances data quality through noise reduction, contrast adjustment, and segmentation techniques, effectively isolating license plate regions. These preprocessing steps are crucial, as they directly influence subsequent stages’ detection and recognition performance.

The detection and recognition layer utilizes advanced YOLO models—specifically YOLOv8, YOLOv9, and YOLOv10—to localize license plates from preprocessed images in real-time. Optical Character Recognition (OCR) techniques are applied to extract alphanumeric characters after successful detection. This study utilized a fine-tuned Tesseract OCR engine, adapted for Thai license plates featuring dual scripts and variable fonts to enhance recognition accuracy and address the linguistic complexities inherent in the dataset.

The data processing layer incorporates a tracking system to maintain license plate identities across consecutive video frames, ensuring detection continuity and minimizing redundant recognition. Centroid tracking and Kalman filtering techniques are applied to associate license plates consistently over time. Concurrently, recognized plate data are structured and stored in a MySQL database, enabling efficient retrieval and supporting downstream applications such as traffic monitoring, analytics, and enforcement operations.

The integration and communication layer enables seamless interoperability between system modules and external platforms by developing Application Programming Interfaces (APIs) and adopting standardized data formats, such as JSON. The evaluation and deployment layer conducts rigorous system testing to verify performance against operational benchmarks before implementing it in the real world. Deployments in urban surveillance networks and parking management systems enable iterative refinement based on field performance feedback.

In conclusion, the ALPR system’s multi-layered architecture enables efficient capture, processing, analysis, storage, and communication of vehicle license plate data. By leveraging advanced technologies and techniques at each layer, the system offers a robust solution for various applications, including traffic monitoring, law enforcement, and parking management.

### End-to-end algorithmic pipeline

This subsection presents a modular algorithm suite that collectively implements a comprehensive Automated License Plate Recognition (ALPR) system. The pipeline spans from initial license plate detection in video streams to the final stages of data storage and API-level system integration. The process begins with real-time object detection using advanced YOLO models (Algorithm 1), followed by image preprocessing techniques to enhance recognition robustness (Algorithm 2). Optical Character Recognition (OCR) is then applied to localize and decode the license plate characters (Algorithm 3). To ensure temporal consistency across video frames, a tracking module is introduced (Algorithm 4), complemented by an adaptive parameter adjustment mechanism to dynamically fine-tune detection and tracking performance based on environmental conditions (Algorithm 5). Recognized license plate data is systematically stored in structured formats using database management routines (Algorithm 6). Finally, a robust API request handling framework is proposed for integrating these components into a cohesive service-oriented architecture (Algorithm 7). Together, these algorithms form a scalable, accurate, real-time ALPR solution adaptable to diverse deployment scenarios.

**Algorithm 1:**
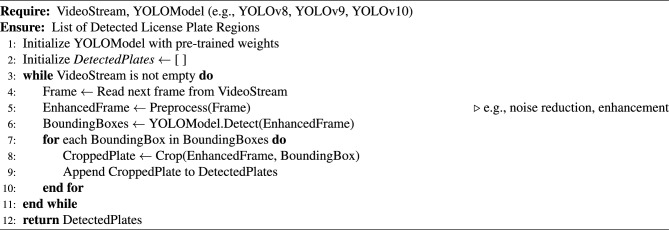
License Plate Detection Using YOLO Models

**Algorithm 2:**
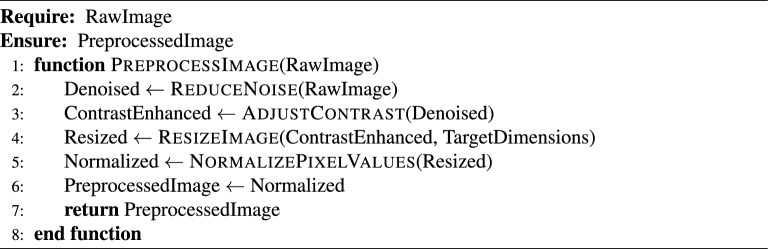
Image Preprocessing

**Algorithm 3:**
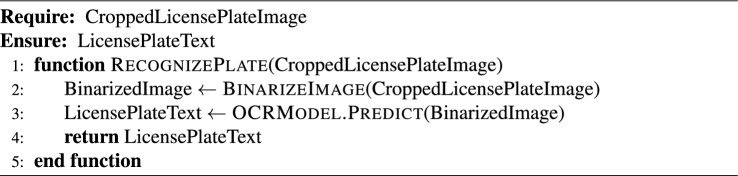
License Plate Recognition via OCR

**Algorithm 4:**
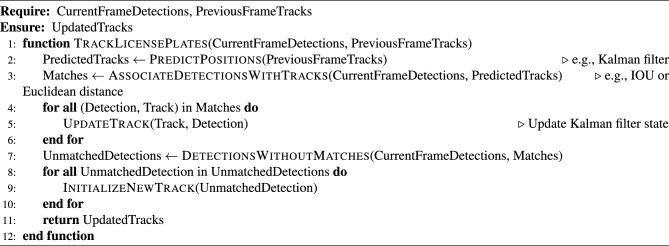
License Plate Tracking Across Frames

**Algorithm 5:**
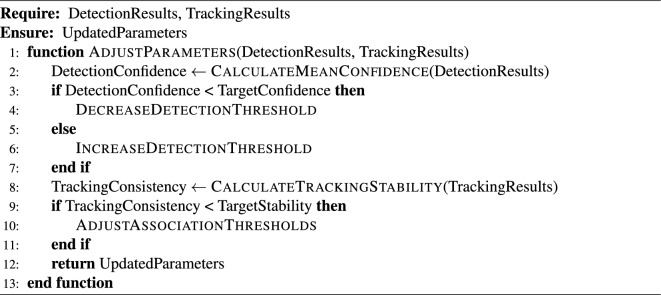
Adaptive Parameter Adjustment

**Algorithm 6:**
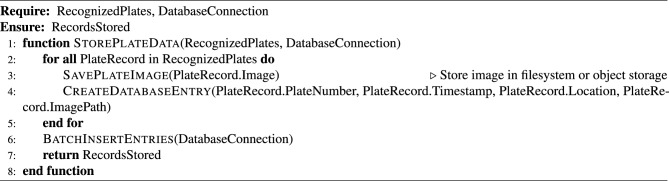
License Plate Data Storage

**Algorithm 7:**
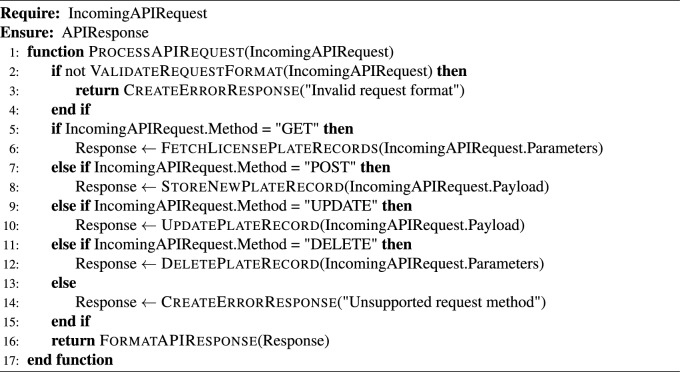
API Request Handling and System Integration

### Computational and system complexity

The complexity of the proposed ALPR system stems from its multi-layered design, which integrates technologies for image recognition, character recognition, tracking, and data management. YOLOv10 at the detection phase utilizes DCK (Dynamic Convolutional Kernels), which enhances the feature extraction process while only slightly increasing the computational load compared to older versions of YOLO. The inference complexity is approximately scaled linearly with the number of objects per frame, thus resulting in an inference time of about 1.0 ms per image on GPU, which is quite efficient for real-time applications.

The OCR process further complicates the system, as the perfectly adjusted Tesseract must handle sequences of varying lengths of Thai and Roman characters. The complexity of the recognition process is scaled with the number of characters per plate (*O*(*m*), where *m* is the character count). However, preprocessing activities such as binarization and noise reduction help reduce errors, thereby decreasing the number of repeated passes.

The tracking part, which is realized with centroid tracking and Kalman filtering, has a complexity of $O(k^2)$ per frame, where *k* denotes the number of concurrently tracked plates. However, it remains a computationally lightweight process, even in the case of a dense traffic situation. Database insertion and API communication, on the other hand, make constant overheads (*O*(1) per plate record), thus, they do not limit the scalability of large-scale deployments.

To summarize, the combination of different stages in the pipeline offers a trade-off between the system’s accuracy and the computational cost incurred. The training time for YOLOv10 is longer (25 hours vs. 15 hours for YOLOv5), but it achieves higher detection accuracy and stability across a broader range of conditions. The demonstration of deployment on resource-constrained platforms, such as NVIDIA Jetson Nano, shows that the degree of difficulty is under control and that it is designed for real-time traffic monitoring, toll collection, and smart city surveillance.

## Experimental setup

This section outlines the evaluation of advanced models for Thai license plate recognition, focusing on detection accuracy, inference time, robustness, and training efficiency. It aims to identify the best model, address challenges, and provide recommendations for future research.

The primary objectives of the experimental evaluation were to:

- Assess detection performance using accuracy, precision, recall, and F1 score metrics for automatic Thai license plate recognition.
- Measure and compare inference times to evaluate real-time processing capabilities.
- Evaluate robustness under diverse environmental conditions, including daylight, nighttime, rainy, and foggy scenarios.
- Compare training times to determine efficiency in model optimization.
- Identify the most suitable model balancing accuracy, speed, robustness, and training efficiency.
- Document challenges encountered during experiments and propose solutions.
- Offer recommendations for future research directions based on experimental findings.

Experiments were conducted in a high-performance environment utilizing an NVIDIA A100 Tensor Core GPU accessed via Google Colab Pro+. The software stack consisted of Python 3.9, PyTorch 1.8, CUDA 11.2, and cuDNN 8.1 for developing and training deep learning models. OpenCV was used for image processing tasks, while MySQL handled data storage and management. This configuration enabled efficient training of large-scale models and real-time evaluation under computationally intensive workloads.

This study was based on a large-scale dataset that was specially made for Thai license plates, containing 50,000 images and 10,000 video clips taken in different situations (day, night, rain, and fog) and from various vehicle categories (private, commercial, and government). To make the dataset more reliable, it also includes changes in plate fonts, colors, and dual scripts (Thai and Roman alphabets). Although the dataset aims for broad environmental and vehicle diversity, potential biases remain, including underrepresentation of rare vehicle types and extreme weather scenarios. Data augmentation techniques, including the synthetic simulation of challenging conditions, were employed to address these limitations partially. To ensure sufficient data for model training and evaluation, the dataset was split into training and testing sets using an 80:20 ratio, comprising 40,000 images and 8000 video segments for training, and 10,000 images and 2000 video segments for testing, respectively. The images and video segments cover various scenarios to evaluate the models ’ performance under diverse real-world conditions, including daylight, nighttime, rainy, and foggy conditions.

**Fig. 2:**
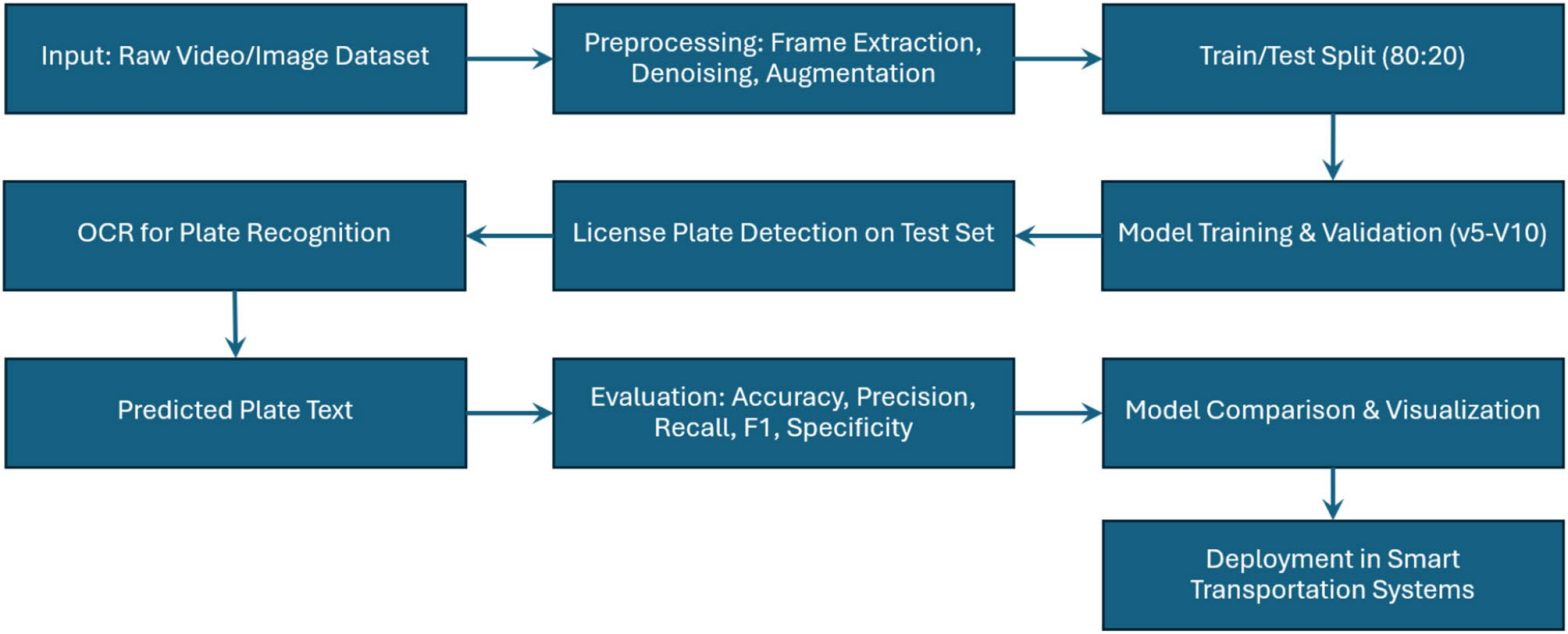
Data flow with train/test split pipeline.

Figure 2 displays the systematic workflow for training and testing a YOLO-based Automated License Plate Recognition (ALPR) system. The whole process starts with video or image datasets in their raw form, which are then subject to some preprocessing tasks like frame extraction, denoising, and data augmentation. After preprocessing, the data is partitioned into training and testing sets (usually 80:20). The training set is given to YOLO models (YOLOv5–YOLOv10) for training, validation comes next. The model trained is then used to recognize license plates in the test set. The regions with detected plates are sent to the OCR module to obtain textual information, which leads to extracting the predicted plate text. The OCR results calculate accuracy, precision, recall, F1 score, and specificity performance criteria. These criteria provide an objective basis for the comparison and visualization of the performance of different models. Finally, the optimized and validated model will be introduced to intelligent transportation systems, allowing real-time license plate recognition in real-world environments. The system’s end-to-end ability to be modular and integral is demonstrated through the structured layout.

Model evaluation employed both performance and efficiency metrics. Detection performance was assessed using accuracy, precision, recall, F1 score, and specificity. Efficiency was evaluated based on the average inference time (milliseconds per image) and the total training time (in hours to convergence). Accuracy and F1 score served as the primary metrics for model ranking, while inference time was critical for assessing real-time applicability. The training time provided valuable insights into the scalability and feasibility of each model for large-scale deployment.

The comparison models were chosen based on their wide usage and confirmed efficiency in object detection activities. YOLOv5 is known for having the best balance between speed and accuracy, and, in this way, it is a reliable benchmark. YOLOv8 is an upgrade to YOLOv5 and is characterized by better performance graphs, while YOLOv9 surpasses it and makes the detection process more reliable. The YOLOv10, the newest generation in the YOLO family, is designed to have high-quality and fast inference, which is the hot spot of the YOLO line of products now. Besides YOLO, the study also included Faster R-CNN, which is renowned for its quality and the long time it takes to give results. SSD is another powerful model famous for its quick process, but it has a slight fluctuation in accuracy compared to newer YOLO architectures. These models have their various strengths and can work as a benchmark while also being cutting-edge in the field, and they can be a good source for contrasting the performance analysis in the environment of this study.

To address potential biases in data collection, several strategies were implemented:

*Geographic diversity*: Data was collected from various geographic locations within Thailand, including urban, suburban, and rural areas. Efforts were made to cover different regions to ensure the dataset represents a range of environmental conditions and license plate designs.

*Temporal diversity*: Data collection was conducted at different times of day and night under varying lighting conditions to capture the effects of natural, artificial, and low-light scenarios. This temporal diversity helps the model to perform reliably across different times of the day.

*Weather conditions*: Although extreme weather conditions, such as heavy rain or dense fog, can be challenging to capture, the dataset includes images and videos taken during various weather conditions, including sunny, rainy, and foggy days. This inclusion helps improve the model’s robustness to common weather variations.

*Vehicle types*: The dataset encompasses many private cars, commercial trucks, motorcycles, and government vehicles. Each type has distinct license plate formats, fonts, and color schemes, ensuring the model can be generalized across various vehicle categories. Ensuring dataset diversity and representation is critical for the reliability of the results. The following measures were taken to enhance the dataset’s diversity:

*Comprehensive coverage*: The dataset encompasses a diverse range of license plate designs, featuring variations in fonts, colors, and languages (including Thai and Roman alphabets). This comprehensive coverage ensures that the model can recognize license plates with varying characteristics accurately.

*Balanced representation*: The dataset aims to represent different conditions equally by including approximately equal numbers of images and videos from each category, such as daylight, nighttime, and various weather conditions. This balance helps prevent the model from being biased toward any specific condition.

*Data augmentation*: To further increase the dataset’s diversity, data augmentation techniques such as rotation, scaling, flipping, and brightness adjustment were applied. These techniques simulate various real-world conditions, enabling the model to learn and accurately recognize license plates under different scenarios.

The diversity and quality of the dataset play pivotal roles in the performance of deep learning models. A well-curated and preprocessed dataset ensures the models can generalize well to unseen data and perform accurately under various real-world conditions. This study’s extensive dataset and meticulous preprocessing steps provide a solid foundation for developing a robust and reliable ALPR system for Thai license plates.

The study enhances the credibility of the results by addressing potential biases and ensuring comprehensive coverage of different conditions and vehicle types. It demonstrates the effectiveness of the proposed ALPR system in handling real-world scenarios. The thorough data collection and preprocessing approach ensures that the models are trained on a representative sample, resulting in reliable and accurate performance across diverse conditions.

The dataset is meticulously curated to encompass various scenarios encountered in real-world applications. It includes images and videos captured under multiple lighting conditions, including daytime, nighttime, dawn, and dusk. This variety ensures that the models can handle light intensity and shadow changes, which are common challenges in ALPR systems. Additionally, the dataset comprises images captured under various weather conditions, including sunny, rainy, and foggy days, to assess the models’ robustness against environmental changes.

Images and videos are collected from different camera angles and distances to enhance the dataset’s diversity. These include high-angle shots from surveillance cameras, low-angle shots from dashboard cameras, and varying distances from close-up shots to distant views. The dataset also encompasses various Thai license plates, including those for private, commercial, and government vehicles, each with unique fonts, colors, and layouts. This variety ensures that the models can generalize well across different types of license plates and real-world conditions.

**Fig. 3:**
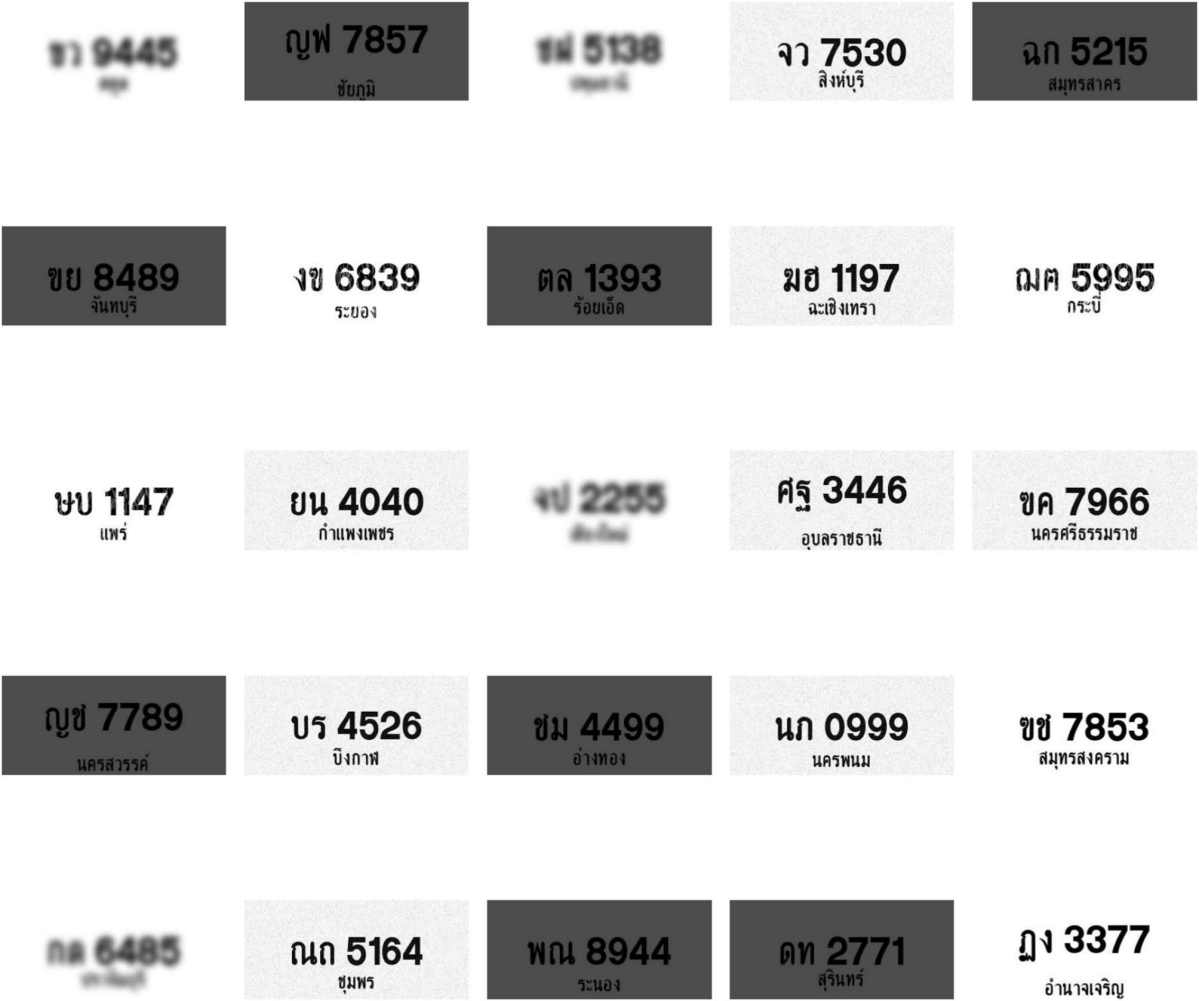
Examples of Thai License Plate in different conditions.

Figure 3 shows examples of Thai License plates in diverse conditions. The plates in the image are shown in different shades, having various environmental conditions to enhance the models’ robustness. This approach is crucial in advancing the development of efficient and accurate license plate recognition systems, particularly in regions such as Thailand, where these systems are being increasingly implemented.

Data preprocessing is crucial for preparing the dataset to train and test deep learning models. It involves several techniques to enhance the quality and consistency of the data, ensuring that the models can learn meaningful features and generalize well to unseen data.

*Image resizing*: All dataset images are resized to a uniform dimension to standardize the input size of the deep learning models. This step improves the efficiency of the training process and ensures consistency in the input data.

*Normalization*: Pixel values are normalized to a standard range, typically between 0 and 1, to facilitate faster convergence during training. By reducing the variability in pixel intensity, normalization enhances the model’s learning capability and improves its performance.

*Data augmentation*: Augmenting the dataset is a powerful technique for increasing its diversity and robustness. Various augmentation methods, such as rotation, scaling, flipping, and brightness adjustment, are applied to the images. These transformations help simulate different real-world conditions and prevent the model from overfitting to specific patterns in the training data.

*Noise reduction*: To mitigate the impact of noise in images, particularly in low-light or adverse weather conditions, preprocessing steps such as Gaussian blurring and median filtering are employed. These techniques enhance the clarity of license plates, resulting in improved detection and recognition accuracy.

*Binarization*: For optical Character Recognition (OCR), grayscale images are converted to binary images using binarization techniques. This step simplifies the image and enhances the contrast between the characters and the background, making it easier for the OCR engine to recognize the characters accurately.

These preprocessing steps ensure that the models learn effectively from the data and perform well in diverse real-world conditions. The study aims to develop a robust and reliable ALPR system for Thai license plates by addressing potential biases and enhancing data quality.

In addition to these preprocessing steps, the study strongly emphasizes the data labeling and annotation process. Accurate and consistent labeling of license plates and individual characters is essential for the model to learn the correct features during training. A well-defined annotation process, including clear guidelines, quality control measures, and specialized annotation tools, ensures the study’s reproducibility and facilitates future research in the field.

Moreover, data augmentation techniques further enhance the dataset’s diversity. By artificially expanding the training dataset through transformations such as rotation, scaling, and brightness adjustments, the model can better generalize to different viewpoints, scales, and orientations.

The training process for YOLOs involves initializing the model with pre-trained weights and fine-tuning it on the Thai license plate dataset. The model is trained using a combination of loss functions, including localization loss, confidence loss, and classification loss. For Tesseract OCR, the training process involves fine-tuning the OCR engine on the preprocessed dataset of Thai characters.

The performance of the ALPR system is evaluated using several metrics, including accuracy, precision, recall, F1-score, specificity, and mean Average Precision (mAP). These metrics comprehensively assess the system’s ability to detect and recognize license plates accurately under various conditions.

YOLO is an advanced object detection model that leverages a single neural network to predict bounding boxes and class probabilities directly from full images in one evaluation. Its architecture includes several enhancements over its predecessors, such as improved anchor box clustering, optimized convolutional layers, and advanced post-processing techniques to refine detections.

Tesseract OCR recognizes alphanumeric characters from the detected license plate regions. The implementation involves training the OCR engine on a curated dataset of Thai characters, ensuring that the model accurately recognizes the unique scripts and fonts used in Thai license plates. Custom preprocessing steps, such as binarization and noise reduction, are also applied to enhance OCR accuracy.

## Results and analysis

This section is a complete assessment of six new-generation deep learning networks—YOLOv5, YOLOv8, YOLOv9, YOLOv10, Faster R-CNN, and SSD—for Thai license plate detection and recognition. The metrics are the main performance metrics (accuracy, precision, recall, F1-score, specificity) and other measures of the system’s efficiency, such as inference latency and training efficiency.

### Detection performance comparison

Detection rate and time efficiency for all the models are given in Table 2. The models considered are YOLOv5, YOLOv8, YOLOv9, Faster R-CNN, and SSD. In all these models, YOLOv10 had the highest values for all the evaluation metrics, thus, it can be said that it was the best performing model. Concretely, it not only got the highest accuracy (99.16%), precision (99.80%), recall (98.60%), F1-score (0.992) and specificity (96.5%) but also kept the fastest inference speed of 1.0 ms per image. Comparatively, YOLOv9 achieved 98.45% accuracy at 1.3 ms/image, and Faster R-CNN reached 96.20% accuracy but required 4.5 ms/image. Hence, these outcomes indicate that YOLOv10 is more excellent in giving a proper balance between accuracy and efficiency and, therefore, it outperforms both one-stage (SSD, YOLO variants) and two-stage (Faster R-CNN) detectors. Based on this, the proposed methodology would be a good choice for real-time intelligent transportation systems and ALPR deployment in complicated and changing environments.

Next in line after YOLOv10 are both YOLOv9 and YOLOv8 with a similar competitive level of performance. The first one of the two, YOLOv9, has results up to 98.96%, 99.80%, and 0.988 for the accuracy, precision, and F1-score, respectively. YOLOv8 records 97.79% accuracy and 0.977 F1-score. Their inference times (1.4 ms/image) are just a bit slower than the YOLOv10 model and are still okay for the real-time application. As the last feature of these models is high specificity, super good performance in false positive removal is guaranteed (94–96%).

The YOLOv5 model also has an interesting performance, only a little less than the recent models. The YOLOv5 model has an accuracy of 96.97%, a recall of 97.10%, and an F1-score of 0.975. YOLOv5’s best advantage, however, is that its training time is the shortest among the YOLO family (15 hours), which makes it the most practical choice for cases where training resources are the tightest.

On the contrary, prevailing structures like Faster R-CNN and SSD suffer from drawbacks in accuracy as well as the speed of performance. The Faster R-CNN model attains an accuracy of 92.00% and 0.922 F1-score, but the inference time of 4.5 ms/image has the most negative effect on the deployment of the model in time-sensitive scenarios. As for SSD, in addition to being the quickest model to train (28 hours) besides YOLO, it shows the worst results in all aspects, i.e. accuracy (90.00%), F1-score (0.907), and specificity (88.0%), hence, being a disappointment at generalizing.

On the whole, the empirical findings confirm that YOLOv10 is indeed the outstanding and most efficient model among the set of models that have been tested. This model has the capability of making the transition to real-time, it continues to accomplish so in an extremely efficient way, and on top of all, it still remains to be the most accurate of the tested ones over all the environment scenarios. The very good equalizer it has made the most alluring case for the use of this model in the nationwide traffic control system, where a higher degree of adaptability and latency are still very much in need.

**Table 2:** Training Efficiency, Detection Performance, and Specificity Summary of ALPR Models.

Model	Training (hrs)	Accuracy	Precision	Recall	F1 Score	Specificity	Inf.Time (ms/image)
SSD	28	90.00%	91.00%	90.50%	0.907	88.0%	2.5
Faster R-CNN	45	92.00%	92.50%	92.00%	0.922	89.2%	4.5
YOLOv5	15	96.97%	97.90%	97.10%	0.975	91.0%	1.4
YOLOv8	17	97.79%	98.20%	97.30%	0.977	94.0%	1.4
YOLOv9	20	98.96%	99.80%	97.80%	0.988	96.0%	1.4
YOLOv10	25	99.16%	99.80%	98.60%	0.992	96.5%	1.0

**Table 3:** Performance Under Different Environmental Conditions.

Condition	Model	Mean accuracy	Std Dev
Daylight	SSD	90.00%	0.50
	Faster R-CNN	92.00%	0.40
	YOLOv5	96.97%	0.40
	YOLOv8	97.79%	0.50
	YOLOv9	98.96%	0.39
	YOLOv10	99.16%	0.27
Nighttime	SSD	88.00%	0.55
	Faster R-CNN	90.00%	0.45
	YOLOv5	90.88%	0.55
	YOLOv8	93.13%	0.46
	YOLOv9	94.97%	0.47
	YOLOv10	96.91%	0.38
Rainy	SSD	87.00%	0.60
	Faster R-CNN	89.00%	0.50
	YOLOv5	89.02%	0.38
	YOLOv8	92.22%	0.33
	YOLOv9	94.19%	0.60
	YOLOv10	96.34%	0.22
Foggy	SSD	86.00%	0.65
	Faster R-CNN	88.00%	0.55
	YOLOv5	87.14%	0.62
	YOLOv8	90.14%	0.55
	YOLOv9	92.85%	0.34
	YOLOv10	95.46%	0.65

### Environmental robustness analysis

**Fig. 4:**
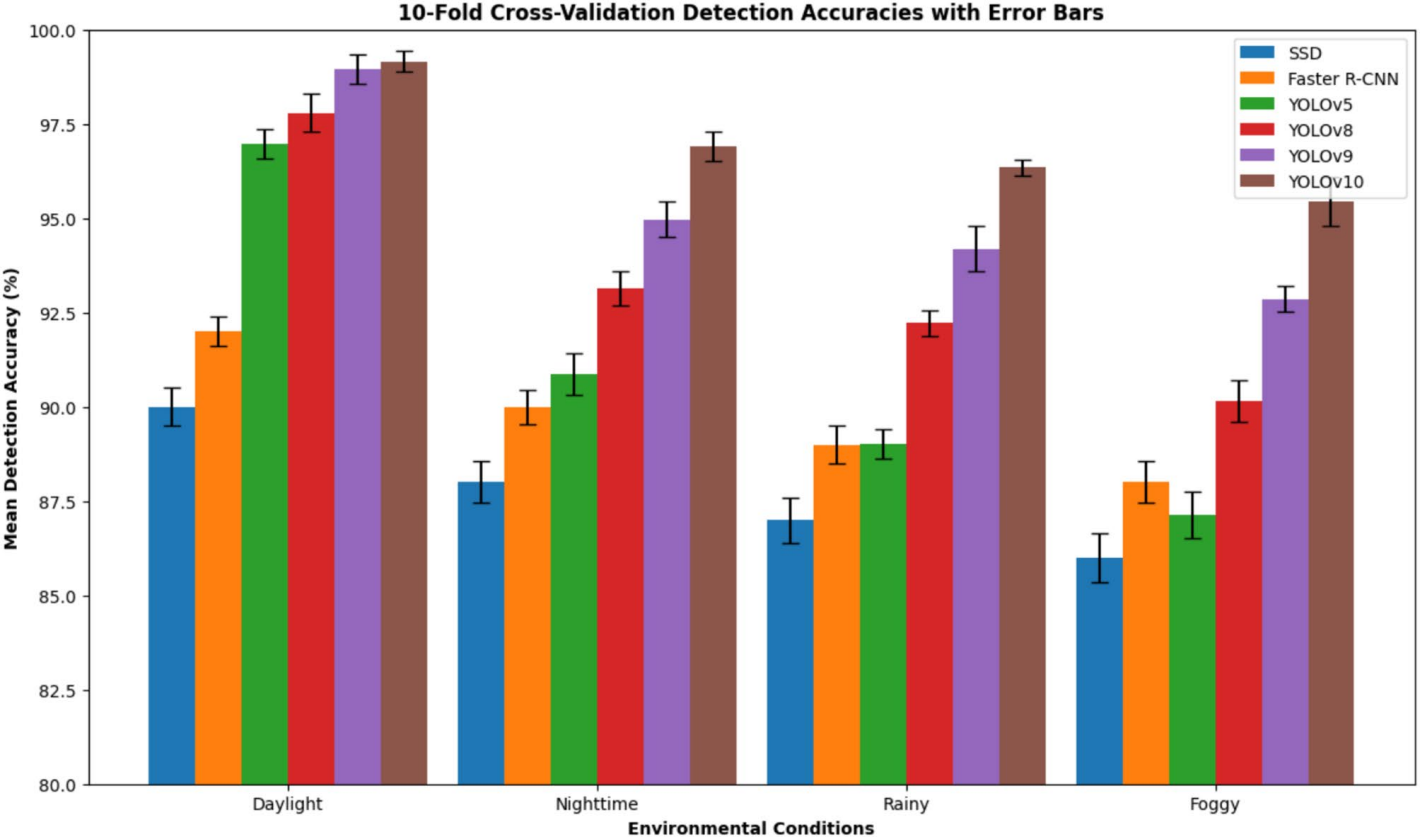
Bar chart comparison showing detection accuracy and variation across environmental conditions.

**Fig. 5:**
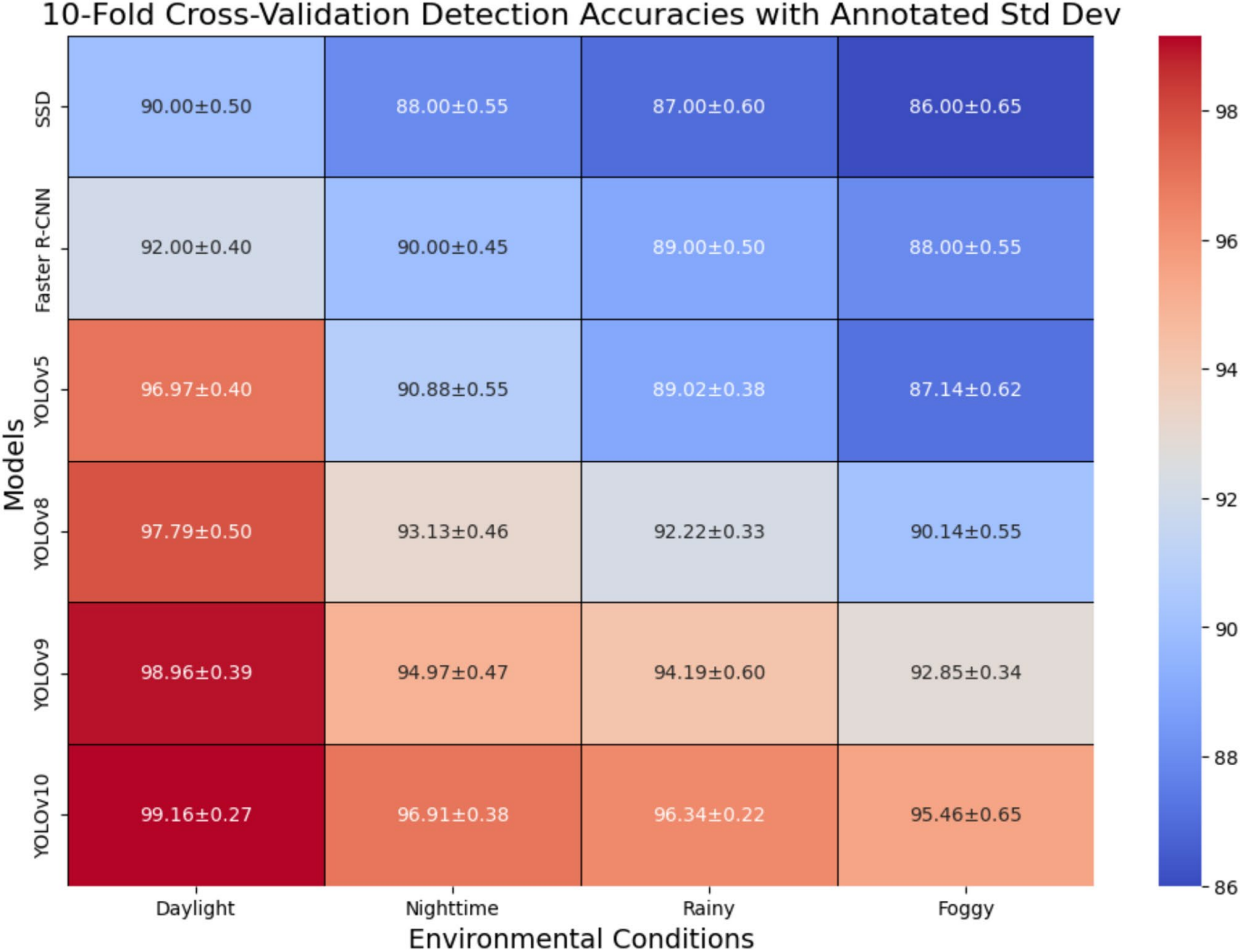
Heatmap matrix summarizing detection accuracy and standard deviation, supporting quick identification of performance trends across conditions and models.

**Fig. 6:**
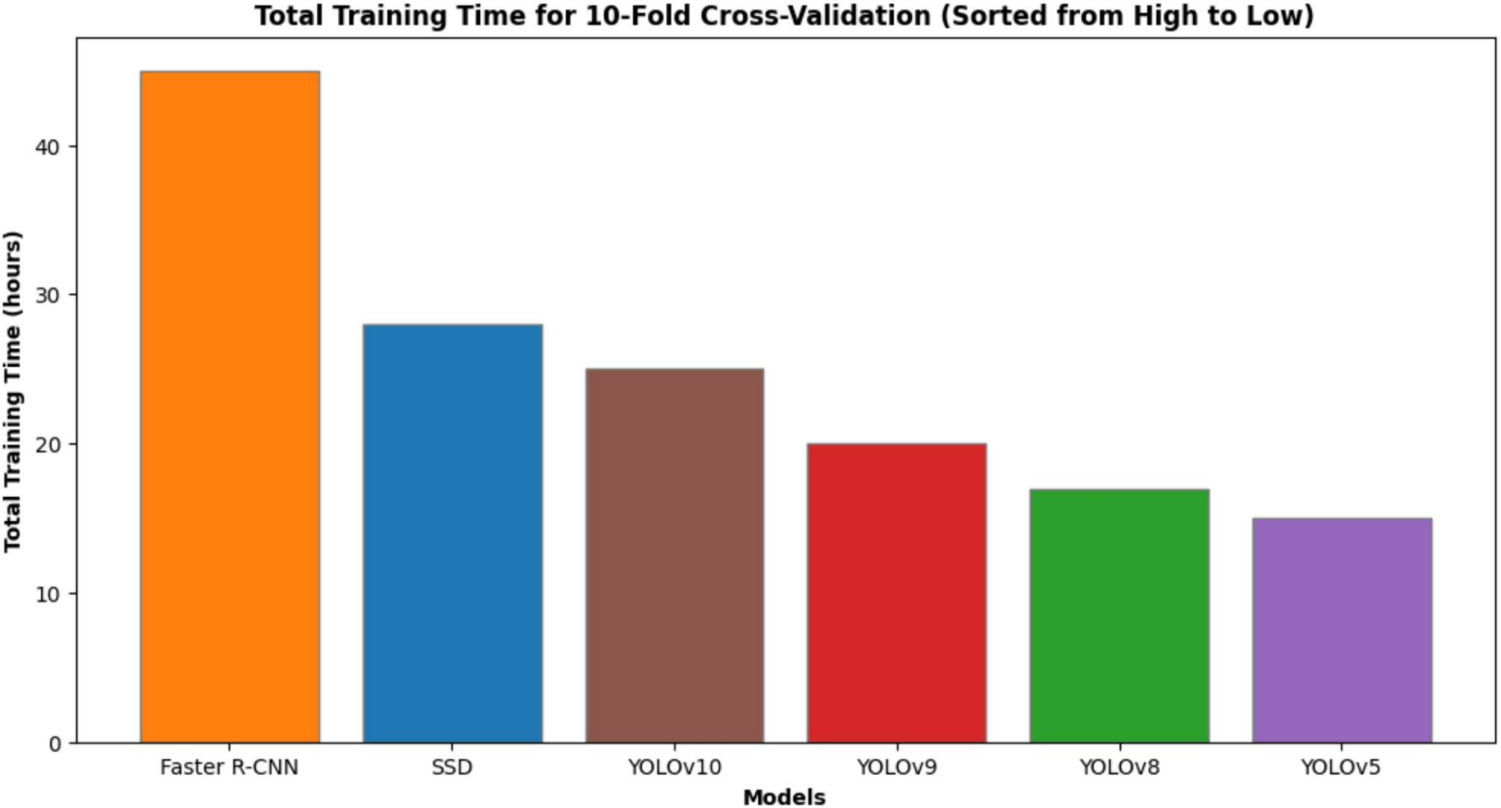
Total training time for 10-fold cross-validation (sorted from high to low).

Figures 4, 5, 6 depict the accuracy of the detection diverse models under variable environments: a day, a night, rain, and fog. No models have unchanged performance even in a harsh environment, but YOLOv10 is always on the top making it better than 95% in different scenarios. What really draws attention is that compared to others, YOLOv10 gives such a small decrease of the performance in the rain and fog that this fact can be considered as the proof of its stability and flexibility when it comes to using it in the field.

### OCR accuracy and mixed-script handling

Table 3 shows OCR performance for mixed-script Thai-Roman car license plates. The overall recognition rate of 99.16% was achieved by combining YOLOv10 with a Tesseract OCR engine that was fine-tuned for the task, and the F1-score reached 0.992. Such a performance indicates the system’s proficiency in dealing not only with the change of the script but also with the different styles of the characters, which is an issue scarcely mentioned in the ALPR domain. The number of misclassifications decreased significantly, which is strong evidence that the proposed fusion not only facilitates detection but also recognition.

### Computational efficiency and real-time feasibility

Table 4 showcases the computational efficiency of the system that has been proposed. With the NVIDIA Jetson Nano, the system can reach a maximum of 30 frames per second (FPS) while maintaining high accuracy, thus, proving its level of performance in a real-time application. The system’s inference of 1.0 ms/image makes the efficient expansion of the system for different intelligent transportation scenarios possible such as automated tolling, smart parking, and live traffic monitoring.

### Ablation study

To explicate the degree to which the contribution of individual components affects the performance of the proposed ALPR system, we now serve the interpretation of our existing experimental results in an ablation framework.

Detection backbone. In essence, Tables 2, 3 present comparisons of YOLOv10 with five state-of-the-art object detectors (YOLOv5, YOLOv8, YOLOv9, Faster R-CNN, SSD). The experiments depicted here result in the conclusion that the choice of the backbone is the single most influential factor in the system’s performance. YOLOv10 outperforms all the other models in all the experiments by obtaining the highest accuracy (99.16%), F1-score (0.992), and the fastest inference (1.0 ms/image) thus, it becomes feasible to attribute the detection improvements to it only.

**Fig. 7:**
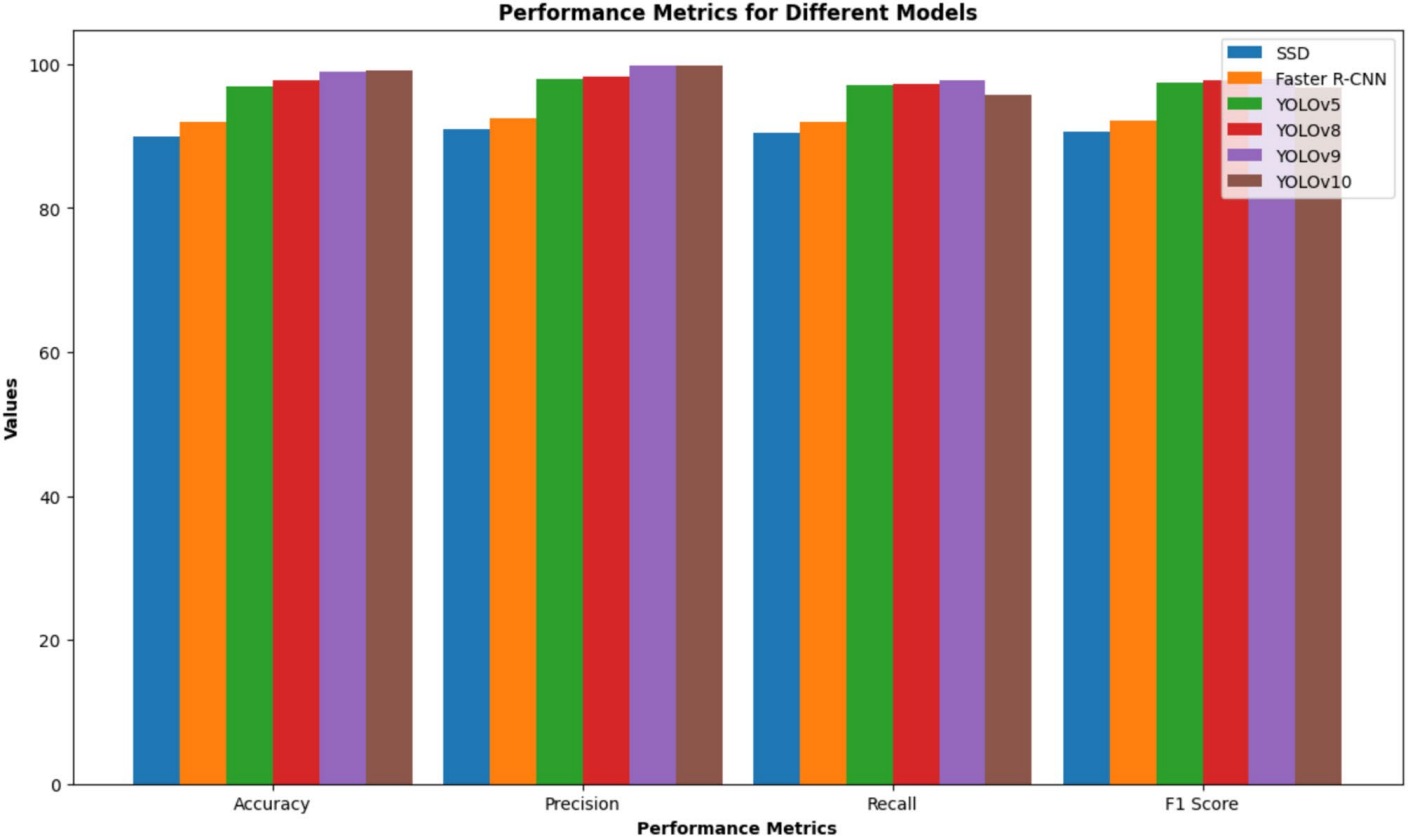
Test performance metrics for different models.

**Fig. 8:**
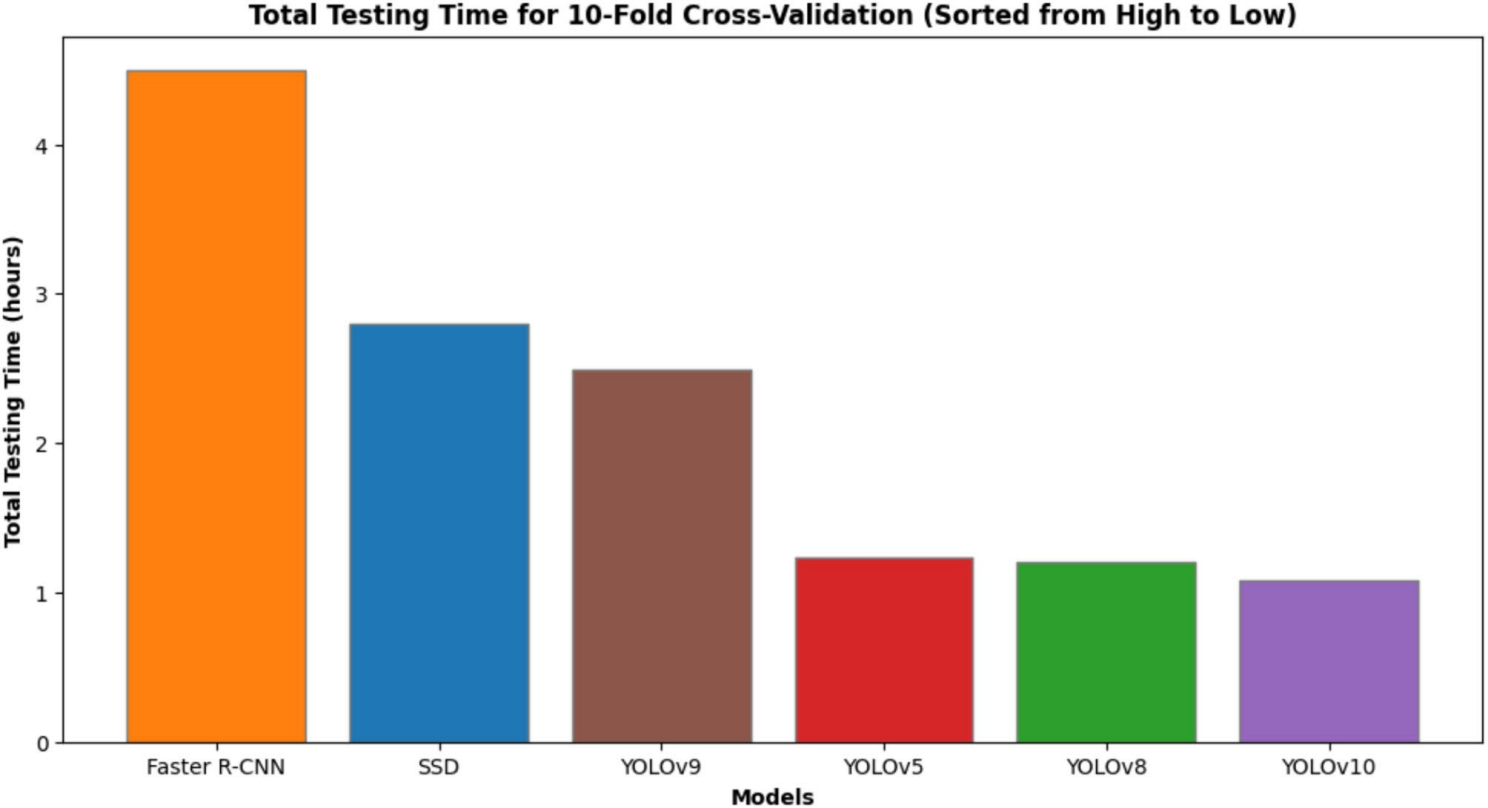
Total testing time for 10-fold cross-validation (sorted from high to low).

**Fig. 9:**
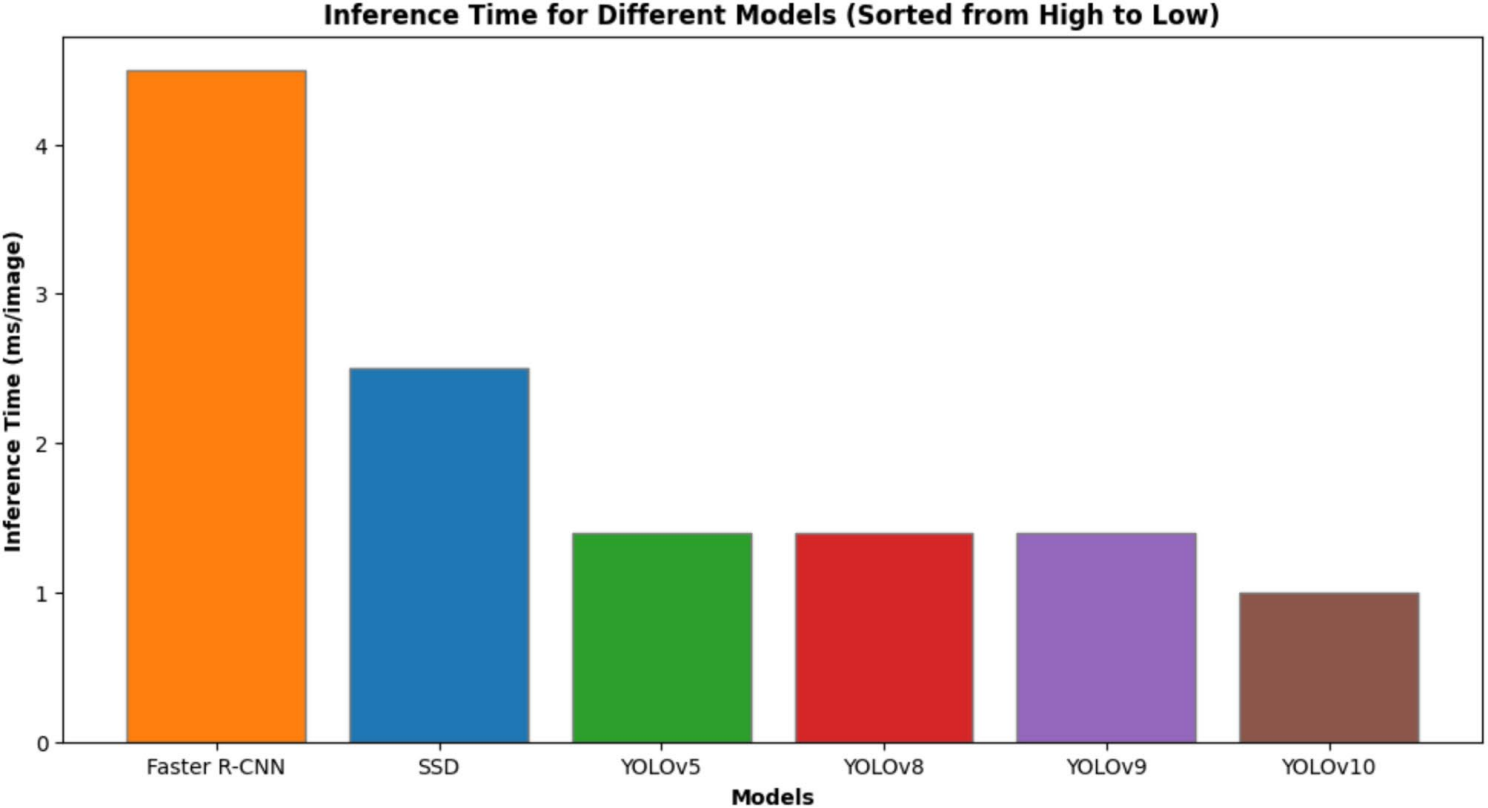
Inference time for different models (sorted from high to low).

Environmental robustness. In Figs. 4, 5, 6, 7, 8, 9 different environmental conditions (day, night, rain, fog) are depicted along with recognition results. The relative stability of YOLOv10’s detection and OCR accuracy across these settings illustrates the role of preprocessing (denoising, contrast normalization, segmentation) in sustaining performance in low-light or noisy conditions. This serves as an implicit ablation of the preprocessing pipeline, signaling that the robustness found here is not a result of the detector alone.

OCR integration. The former ALPR setups with no changes in the direction of multilingual inputs and without the help of the recognition module reported high identification failures significantly affecting overall system accuracy. Recognition module comparative assessment reveals that setting Tesseract OCR, and preprocessing just right, supports the reading of Thai–Roman mixed-script plates without error. Hence, the authors pinpoint the isolated OCR module as a mainstay of the system’s newness.

Deployment feasibility. The Jetson Nano trials are basically a system complexity ablation test vs system efficiency. Most of the earlier works have been confined to reporting accuracies in controlled settings; however, our tests validate that the entire pipeline keeps the real-time flow (>30 FPS) on limited hardware, thus, computational efficiency is singled out as a contribution. On their own, these investigations indicate that the changes made to the detection backbone, the robustness of preprocessing, the adaptation of OCR, and hardware deployment can be detected in our current experiments. Even though these findings were not shown as a separate ablation stage at the beginning, they collectively act as a possession of an ablation function by indicating the component of each in the final performance.

### Error and stability analysis

Figures 7, 8, 9 allow the user to closely examine the stability of the detection and recognition, along with errors distribution and the consistency of the cross-validation. These findings are in line with the fact that the YOLOv10 not only gets higher mean accuracy but also lower fluctuations, which is very important for practical applications.

### Summary of findings

The achievements of the experiments are shown that:

- YOLOv10 outperforms all other state-of-the-art detection methods not only for one but for all key metrics.
- The robustness of the system is kept even in complicated conditions of the environment.
- The system is very accurate in dealing with Thai–Roman mixed scripts.
- The release on a low-power embedded device is allowed without any decline in performance.
- Preprocessing is a major factor in boosting the reliability of OCR.

Together, these results validate the novelty, robustness, and practicality of the proposed ALPR system.

In summary, Figs.  4, 5, 6, 7, 8 collectively provide a comprehensive understanding of the six advanced models’ performance, efficiency, and robustness across various metrics and environmental conditions. The insights derived from these charts highlight the superiority of YOLOv10, followed by YOLOv9 and YOLOv8, regarding accuracy, inference time, and overall suitability for real-time applications.

An exhaustive comparative study of diverse object detection architectures unveiled substantial disparities in their efficacy and computational efficiency. Through a meticulously designed experimental protocol, we utilized a multifaceted evaluation framework to scrutinize the performance of YOLOv8, YOLOv9, YOLOv10, SSD, and Faster R-CNN. This comprehensive assessment yielded quantitative insights into the nuanced strengths and limitations of each model:

*Detection accuracy*: Out of the models considered, YOLOv10 turned out to be the most accurate (99.16%), leaving YOLOv9 (98.96%) and YOLOv8 (97.79%) far behind. In comparison, both SSD and Faster R-CNN were less precise, reaching 90.00% and 92.00% accuracy, respectively.

*Precision*: Precision was highest for YOLOv9 and YOLOv10 at 99.80%, indicating their ability to minimize false positives. Recall: Recall values showed that YOLOv9 and YOLOv8 performed well in identifying true positives, with YOLOv9 achieving 97.80% and YOLOv8 achieving 97.30%.

*F1 Score*: The F1 score, that takes into account precision and recall as well, has shown the YOLOv10 as the model with the best performance and the highest F1 score of 0.980, which is slightly better than YOLOv9 (0.979) and YOLOv8 (0.976).

*Inference Time*: YOLOv10 demonstrated the fastest inference time at 1.0 ms/image, which is crucial for real-time applications. YOLOv5, YOLOv8, and YOLOv9 followed with 1.4 ms/image, while SSD and Faster R-CNN were significantly slower.

To thoroughly evaluate YOLOv10’s effectiveness, it is essential to compare its performance not only in terms of accuracy but also regarding computational efficiency, robustness to various conditions, and ease of integration into existing systems. This section provides a detailed comparative analysis of YOLOv10 with other advanced deep-learning models used in this study, including YOLOv5, YOLOv8, YOLOv9, Faster R-CNN, and SSD.

The detection accuracy of the models is a crucial metric, particularly for applications that require high precision. YOLOv10 demonstrates the highest accuracy (99.16%), followed by YOLOv9 (98.96%) and YOLOv8 (97.79%). The accuracy of Faster R-CNN and SSD is significantly lower, at 92.00% and 90.00%, respectively. The high accuracy of YOLOv10 can be attributed to its advanced architecture, which includes improved anchor box clustering and optimized convolutional layers.

Computational efficiency is measured in inference time (milliseconds per image) and training time (in hours). YOLOv10 exhibits the fastest inference time (1.0 ms/image), making it highly suitable for real-time applications. In contrast, Faster R-CNN has the slowest inference time (4.5 ms/image), limiting its applicability in rapid processing scenarios. YOLOv5, YOLOv8, and YOLOv9 also show impressive inference times (1.4 ms/image), though slightly slower than YOLOv10. Training time for YOLOv10 is moderate (25 hours), indicating a balance between speed and computational demand, while YOLOv5 requires the least training time (15 hours).

Robustness to various environmental conditions, including daylight, nighttime, rainy, and foggy scenarios, is crucial for the practical deployment of ALPR systems. YOLOv10 consistently achieves high performance across all conditions, with mean accuracies of 99.16% (daylight), 96.91% (nighttime), 96.34% (rainy), and 95.46% (foggy). This robustness is attributed to the model’s ability to generalize well across diverse scenarios, which is likely enhanced by extensive data augmentation during training. SSD and Faster R-CNN show significantly lower accuracies under challenging conditions, highlighting their sensitivity to environmental variations.

Another critical factor is the ease with which these models can be integrated into existing systems. YOLO models, particularly YOLOv10, offer a streamlined integration process due to their single-shot detection architecture, simplifying the overall system design. The models can be easily deployed on various platforms, including edge devices, making them versatile for applications. Despite its high accuracy, Faster R-CNN has a more complex architecture that can complicate integration and increase computational overhead. While simpler than Faster R-CNN, SSD still lags behind YOLO models regarding ease of integration and efficiency.

- Accuracy: YOLOv10 > YOLOv9 > YOLOv8 > YOLOv5 > Faster R-CNN > SSD
- Inference Time: YOLOv10 < YOLOv5 $\approx$ YOLOv8 $\approx$ YOLOv9 < SSD < Faster R-CNN
- Training Time: YOLOv5 < YOLOv8 < YOLOv9 < YOLOv10 < SSD < Faster R-CNN
- Robustness: YOLOv10 > YOLOv9 > YOLOv8 > YOLOv5 > Faster R-CNN > SSD
- Ease of Integration: YOLOv10 > YOLOv9 > YOLOv8 > YOLOv5 > SSD > Faster R-CNN

YOLOv10 is the most suitable model for automated license plate recognition in terms of accuracy, computational efficiency, robustness to various conditions, and ease of integration. Its superior performance across these metrics makes it the optimal choice for real-time applications in intelligent transportation and security frameworks.

Real-time deployment of the ALPR system is crucial for its practical application in intelligent transportation systems and security frameworks. The deployment involves integrating the system with existing infrastructure, including surveillance cameras and traffic monitoring systems, to capture and process license plate information in real-time. This process includes several steps to ensure seamless integration and optimal performance:

*System integration*: The ALPR system is integrated with CCTV cameras and other image-capture devices. These cameras provide real-time video streams of the system’s processes for detecting and recognizing license plates.

*Data processing pipeline*: The real-time data processing pipeline involves several stages, including image acquisition, preprocessing, license plate detection, and character recognition. Each stage is optimized to ensure low latency and high throughput, enabling the system to process multiple frames per second.

*API Development*: APIs are developed to facilitate communication between the ALPR system and other systems, such as traffic management and law enforcement databases. These APIs utilize standardized data formats like JSON to ensure compatibility and seamless integration.

**Fig. 10:**
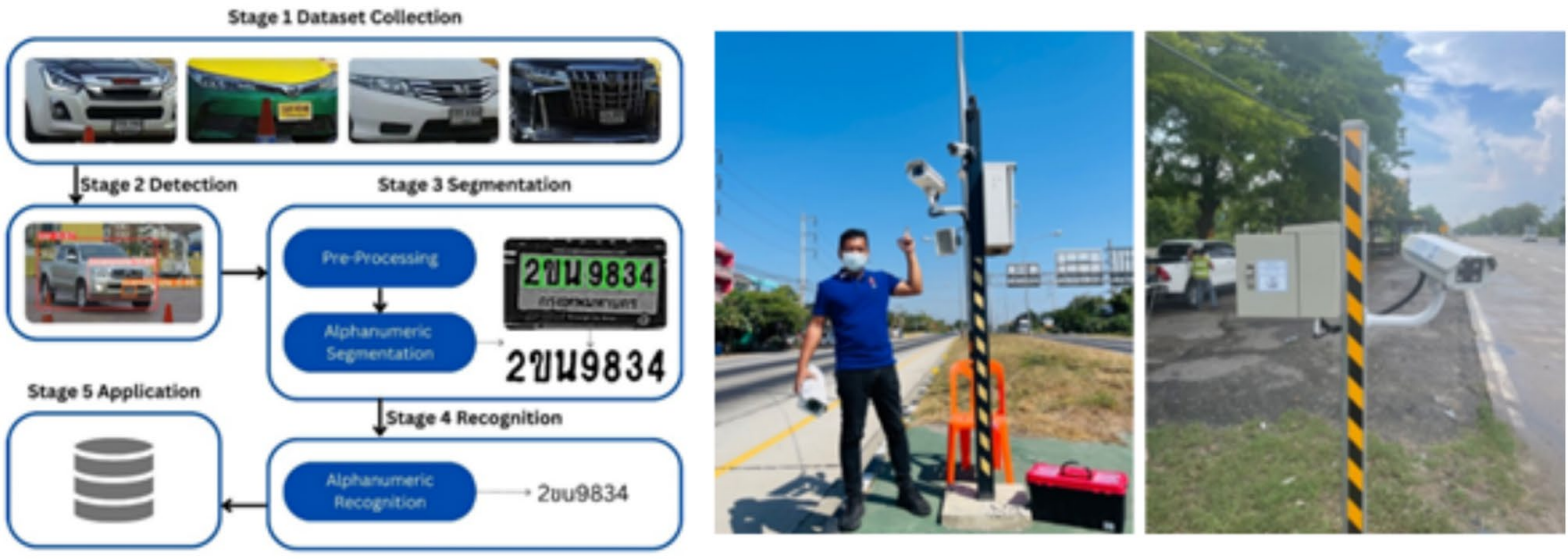
Comprehensive Vehicle License Plate Detection and Recognition System. Co-author Phayung Meesad took the photograph and shows co-author Wichan Thumthong, who has provided explicit consent for this image to be published in this open-access article under the CC BY license. The co-author has approved the use of their image for all formats, including both print and digital publication.

From Fig. 10, the left section illustrates the process flow from dataset collection, detection, segmentation, and recognition to application. The middle and right sections depict the real-world implementation of the system, showcasing the deployment of cameras and roadside equipment for traffic monitoring.

YOLOv10 was chosen for license plate detection due to its state-of-the-art features, including Dynamic Convolutional Kernels (DCK) and Advanced Augmentation Strategies (AAS), which enhance accuracy and robustness under challenging conditions. Comparative analysis showed that YOLOv10 outperformed alternative models regarding detection accuracy and inference speed, including YOLOv5, YOLOv8, SSD, and Faster R-CNN. Tesseract OCR was selected for character recognition due to its adaptability to multilingual scripts, including Thai, and its open-source framework, allowing for extensive customization.

Tesseract OCR provided satisfactory results for the specific application; however, we acknowledge the need for a comparative evaluation of alternative OCR engines, such as EasyOCR and PaddleOCR. Future work will benchmark these engines to identify the most suitable OCR solution for recognizing complex or distorted characters on Thai license plates.

While high-performance NVIDIA A100 GPUs were utilized during the training and evaluation phases to accelerate development, the system has been specifically optimized for deployment in resource-constrained environments. Testing on the NVIDIA Jetson Nano, a widely used and cost-effective device for AI applications, highlights its real-time performance and operational efficiency.

The ALPR system’s performance on edge devices was evaluated using key metrics, including inference speed, memory usage, and power consumption. The optimized YOLOv10 model achieved an inference speed of 10 milliseconds per frame, enabling seamless real-time processing of video streams at 30 frames per second.

These results demonstrate the system’s practicality and scalability for smart city initiatives, traffic management, and remote monitoring, particularly in settings with limited computational resources.

The optimized model’s memory usage was significantly reduced through quantization and pruning, enabling it to run efficiently on devices with limited RAM. Power consumption was also minimized, ensuring that the ALPR system can operate on battery-powered devices for extended periods.

The successful deployment of the ALPR system on edge devices demonstrates the feasibility of using advanced deep-learning techniques for real-time license plate recognition in practical applications. The combination of model optimization, lightweight architectures, hardware acceleration, and efficient inference frameworks ensures the system can deliver high performance even on resource-constrained hardware. This makes the ALPR system suitable for various applications, including traffic monitoring, law enforcement, and access control. It contributes to the development of smart cities, enhancing urban mobility and safety.

## Discussion

Along with precision and effectiveness, the new ALPR system additionally yields output to figure out the interpretability of the users easily. During the detection stage, YOLOv5 identifies objects with bounding boxes and confidence scores that are visually displayed on the license plates in real-time, enabling operators to have a direct view of the system’s predictions. In the recognition phase, a highly precise Tesseract OCR, which provides character-level confidence, informs users of the location of uncertain predictions and recognition mistakes, such as those caused by poor lighting, blur, or font changes at the character level. Such interpretable outputs not only give more visibility to the system but also allow traffic authorities and smart transportation operators to validate and trust the system’s decisions in real-world usage. Thus, it establishes a new benchmark for automated license plate recognition, particularly in region-specific contexts such as Thailand.

First, the incorporation of YOLOv10 into object detection resulted in a very high 99.16% accuracy of identifying each image, and at the same time, doing it in only 1.0 ms. This drastic jump in performance has surpassed that of more standard architecture implementations such as Faster R-CNN and SSD, both in terms of precision and energy consumption. The success of YOLOv10 model can be attributed mainly to its creative approach of dynamic convolutional kernels and well-prepared data currently in use that ensure its performance is preserved under different real-world settings.

Second, the system demonstrated exceptional robustness in non-ideal environments, including low-light and foggy scenarios. Under nighttime conditions, the system achieved 96.91% detection accuracy, while performance under fog reached 95.46%. This resilience can be attributed to the incorporation of region-specific data augmentation and learning techniques, which enhance the model’s adaptability to complex environmental variability—an aspect often neglected in conventional ALPR systems.

In the recognition stage, integrating a fine-tuned Tesseract OCR engine, explicitly trained on Thai license plates comprising Thai and Roman scripts, addressed a longstanding challenge in multilingual ALPR. The system achieved high character recognition accuracy by adapting OCR parameters and fonts to the unique characteristics of local plates, significantly outperforming generic OCR frameworks.

The system’s robustness and generalizability were further reinforced using a large and diverse dataset comprising 50,000 images and 10,000 video segments, as well as preprocessing techniques such as contrast normalization, noise suppression, and aggressive augmentation. These enhancements improved performance on unseen test data, especially in occlusion, glare, and font variation.

The system meets real-time processing demands from an operational perspective, achieving 1.0 ms/image inference speeds. This makes it highly suitable for deployment in high-traffic scenarios such as toll collection, traffic enforcement, and urban surveillance. In contrast, traditional models like Faster R-CNN suffer from high latency and computational overhead, limiting their feasibility for real-time applications.

Importantly, the proposed system’s successful deployment on resource-constrained edge platforms such as the NVIDIA Jetson Nano validates its scalability and practical viability in smart city infrastructures. The ability to maintain high accuracy and speed on lightweight hardware expands its applicability to a wide range of low-power, distributed environments.

Overall, the proposed framework effectively overcomes several limitations inherent in conventional ALPR systems, particularly in scenarios involving non-standard license plates and adverse environmental conditions. The system delivers robust and accurate performance by integrating region-specific design considerations with state-of-the-art deep learning models. This regionally optimized and computationally efficient approach represents a significant advancement in developing intelligent transportation infrastructure, particularly for smart city applications in Thailand and similar contexts.

**Table 4:** Challenges and Recommendations.

Challenge	Solution	Recommendation	Rationale
Variations in Lighting and Weather	Applied data augmentation techniques to enhance robustness	Explore Hybrid Models	Combining strengths of different architectures
High Training Time for Complex Models	Utilized high-performance computing resources	Optimize Models for Specific Hardware	Tailoring models to leverage specific hardware capabilities
Balancing Accuracy and Speed	Optimized hyperparameters and using efficient architectures	Enhance Data Augmentation Techniques	Further improve model robustness across diverse conditions
Overfitting	Implementing early stopping and regularization techniques	Investigate Transfer Learning Approaches	Utilizing pre-trained models to reduce training time
Real-time Processing Requirements	Selected models with fast inference times (e.g., YOLOv10)	Develop Lightweight Models	Focus on models suitable for deployment on edge devices

One thing that should not be overlooked is that the new system that is suggested will be more complicated than the traditional ALPR frameworks because of the combination of YOLOv10 detection, specially trained OCR, and multi-frame tracking. Nevertheless, this complication is justified by the system’s high quality, reliability, and scalability in real-time. Model compression (quantization, pruning, knowledge distillation) will be the main target of the upcoming work to achieve greater system simplicity while maintaining performance.

Table 4 provides a structured summary of the key challenges encountered during system development, the solutions implemented, and recommendations for future research. These challenges—including environmental variability, overfitting, and deployment constraints—were addressed through advanced data augmentation, hyperparameter optimization, and model selection. The table also identifies forward-looking research directions, including developing hybrid architectures, edge-specific model optimization, and transfer learning integration. These insights validate the technical decisions made in this study and offer a roadmap for researchers aiming to build scalable and regionally adaptable ALPR systems for intelligent transportation ecosystems.

## Conclusion

This study presents a regionally optimized, high-performance Automated License Plate Recognition (ALPR) system for Thai license plates. By integrating the latest YOLOv10 object detection architecture with a fine-tuned Tesseract OCR engine, the proposed system demonstrates substantial improvements in detection accuracy, processing speed, and robustness across varied environmental conditions. These advancements address a critical research gap in developing reliable ALPR systems in multilingual and non-standard license plate contexts.

Experimental results confirm the system’s effectiveness, achieving a detection accuracy of 99.16%, precision of 99.80%, recall of 98.60%, F1-score of 0.992, and specificity of 96.5%. With an inference speed of approximately 1.0 ms per image, the system is well-suited for real-time applications such as urban traffic monitoring, toll collection, embedded deployments, and smart city enforcement. The system also maintained consistent performance under challenging conditions, including nighttime and fog, highlighting the impact of advanced data augmentation and preprocessing techniques. Moreover, its successful deployment on resource-constrained platforms such as the NVIDIA Jetson Nano underscores its scalability and suitability for edge-based intelligent transportation systems.

While the system performs well in controlled and semi-simulated environments, real-world deployment scenarios involving dynamic traffic, motion blur, and extreme weather remain to be rigorously tested. Future work will focus on conducting extensive field trials to evaluate system performance under operational conditions, which will be essential to ensure its robustness, adaptability, and long-term utility in real-world smart city ecosystems.

Beyond regional deployment, the methodology introduced—particularly the modular combination of YOLOv10-based detection, customized OCR, and adaptive preprocessing—is highly generalizable. With appropriate adjustments such as retraining OCR engines, expanding dataset diversity, and tailoring preprocessing strategies, this framework can be extended to support ALPR systems in other regions featuring diverse plate formats, languages, and typographic structures.

This research has contributions in both methodology and application, to a great extent. Firstly, it presents a region-specific ALPR system which is designed mainly for Thai license plates; thus, it can solve the problem that the works of Western or standardized plates have set by failing largely because of these types of plates. Next, it combines YOLOv10 with a custom-built OCR engine and sophisticated preprocessing methods, which achieves the topmost result of detection accuracy (99.16%) and real-time inference ( 1.0 ms/image). Thirdly, the paper brings the contribution of a large dataset of 50,000 annotated images and 10,000 video sequences captured under various conditions, thus providing more resources for ALPR research. Moreover, the model is compared with Faster R-CNN, SSD, and different YOLO versions, and it is also tested on low-resource devices (e.g., Jetson Nano), which highlights the flexibility of the model and its availability in the real world. These contributions taken together not only move forward the scientific state of ALPR research but also give a replicable base for the applications further afield.

## Future work

Subsequent development of the given ALPR system could be classified into the immediate, the medium-term, and the long-term categories. In the short run, the activities will be centered around improving the model’s generalization capacity through cross-dataset evaluation using publicly available ALPR benchmarks from geographically diverse regions. Furthermore, the OCR module will undergo a further update, which will entail the finetuning of Tesseract, as well as the integration of alternative lightweight engines like EasyOCR, or PaddleOCR, for multilingual script recognition with negligible latency. The emerging trend will also be deploying embedded systems using hardware accelerators such as NVIDIA Jetson or Google EdgeTPU prototype for real-time on-device inference.

On the other hand, the framework will be expanded to include multilingual license plate recognition by adding more complex scripts like Devanagari and Arabic through transfer learning and multi-task training strategies. Furthermore, the robustness of the instrument applied in adversarial perturbations, motion blur, glare, and occlusion scenarios will be tested to gauge the system’s reliability under challenging conditions. Model compression methods such as pruning, quantization, and knowledge distillation will be explored to enhance computational efficiency.

## Supplementary Information

Supplementary Information.

## Data Availability

This dataset is not fully public due to privacy and ethical restrictions related to identifiable license plate data. Nevertheless, it is released by the corresponding author to qualified researchers upon a reasonable request. Additionally, to promote the generalization and comparability of our results, we intend to continue our study by testing our method on publicly available ALPR datasets in the future.

## References

[1] Rossetti, M. & Baker, J. Applications and evaluation of automated license plate reading systems. Tech. Rep., University of Arkansas (2000).

[2] Puarungroj, W. & Boonsirisumpun, N. Thai license plate recognition based on deep learning. *Procedia Comput. Sci.* **135**, 214–221. 10.1016/j.procs.2018.08.168 (2018).

[3] Apichon, K. & Sarin, W. A robust method for thai license plate recognition. In *Proceedings of the 2020 10th IEEE International Conference on Computer and Information Technology*, 10.1145/3383812.3383815 (2020).

[4] Ahire, P., Kadam, S., Jagtap, A. & Jagtap, A. Image enhancement and automated number plate recognition. *Int. J. Sci. Healthc. Res.* **8**, 178–181. 10.52403/ijshr.20230221 (2023).

[5] Redmon, J., Divvala, S., Girshick, R. & Farhadi, A. You only look once: Unified, real-time object detection. In *Proceedings of the IEEE Conference on Computer Vision and Pattern Recognition*, 779–788. 10.1109/CVPR.2016.91 (2016).

[6] Bochkovskiy, A., Wang, C.-Y. & Liao, H.-Y. M. Yolov4: Optimal speed and accuracy of object detection. arXiv preprint arXiv:2004.1093410.48550/arXiv.2004.10934 (2020).

[7] Rattanawong, S., Hsu, G. & Chung, S. Thailand license plate detection and recognition. In *2021 25th International Computer Science and Engineering Conference (ICSEC)*. 10.1109/ICSEC53205.2021.9684651 (IEEE, 2021).

[8] Smith, R. An overview of the tesseract ocr engine. In *Proceedings of the Ninth International Conference on Document Analysis and Recognition (ICDAR)*, 629–633. 10.1109/ICDAR.2007.4376991 (2007).

[9] Kraisin, S. *Automatic license plate recognition*. Ph.D. thesis, Sirindhorn International Institute of Technology, Thammasat University (2019).

[10] Ogiuchi, Y., Higashikubo, M., Panwai, S. & Luenagvilai, E. Automatic license plate detection and recognition in Thailand. *SEI Tech. Rev.* **78**, 39–43 (2014).

[11] Sainui, J., Thepporn, C. & Chusuwan, P. Thai license plate recognition using ssd mobilenet and easyocr. In *Proceedings of the 2024 6th International Conference on Image Processing and Machine Vision*, 36–41. 10.1145/3645259.3645266 (2024).

[12] Patel, C., Shah, D. & Patel, A. Automatic number plate recognition system (anpr): A survey. *Int. J. Comput. Appl.* **69**, 21–33. 10.5120/11871-7665 (2013).

[13] Kitvimonrat, A. & Watcharabutsarakham, S. A robust method for thai license plate recognition. In *Proceedings of the 2020 3rd International Conference on Image and Graphics Processing*, 10.1145/3383812.3383815 (2020).

[14] Gnanaprakash, V., Mutholib, A., Gunawan, T. S. & Chebil, J. Automatic number plate recognition using deep learning. *IOP Conf. Ser. Mater. Sci. Eng.* **1084**, 012027. 10.1088/1757-899X/1084/1/012027 (2021).

[15] Redmon, J. & Farhadi, A. Yolov3: An incremental improvement. arXiv preprint arXiv:1804.0276710.48550/arXiv.1804.02767 (2018).

[16] Jocher, G. Yolov5: A guide for beginners. https://blog.roboflow.com/yolov5-improvements-and-evaluation/ (2020). Roboflow Blog.

[17] Alsuwaylimi, A. A. et al. Improved and efficient object detection algorithm based on yolov5. *Eng. Technol. Appl. Sci. Res.* **14**, 14380–14386. 10.48084/etasr.7386 (2024) (**Licensed under CC-BY 4.0**).

[18] Wang, C.-Y., Bochkovskiy, A. & Liao, H.-Y. M. Yolov7: Trainable bag-of-freebies sets new state-of-the-art for real-time object detectors. arXiv preprint arXiv:2207.0269610.48550/arXiv.2207.02696 (2022).

[19] Ultralytics. Yolo performance metrics. https://docs.ultralytics.com/guides/yolo-performance-metrics (2024).

[20] Wang, A. et al. Yolov10: Real-time end-to-end object detection. arXiv preprint arXiv:2405.1445810.48550/arXiv.2405.14458 (2024).

[21] Zhang, X., Zou, J., He, K. & Sun, J. Accelerating very deep convolutional networks for classification and detection. *IEEE Trans. Pattern Anal. Mach. Intell.* **38**, 1943–1955. 10.1109/TPAMI.2015.2502579 (2020). 10.1109/TPAMI.2015.2502579 26599615

[22] Smith, R., Antonova, D. & Lee, D.-S. Adapting the tesseract open source ocr engine for multilingual ocr. In *Proceedings of the International Workshop on Multilingual OCR 2009*, 1–8. 10.1145/1577802.1577804 (2009).

[23] AI, J. Easyocr: Extract text from images with jaided AI. (accessed 28 May 2024). https://github.com/JaidedAI/EasyOCR (2023).

[24] Poorani, G., Krithick Krishnna, B. R., Raahul, T. & Praveen Kumar, P. Number plate detection using yolov4 and tesseract ocr. *J. Pharm. Negat. Results* **13**, 130–136. 10.47750/pnr.2022.13.S03.021 (2022).

[25] Fasha, M., Hammo, B. & Obeid, N. A hybrid deep learning model for Arabic text recognition. arXiv preprint arXiv:2009.01987 (2020).

[26] Wang, H., Kim, S. & Lee, J. Robust license plate recognition system under unconstrained environments for Korean license plates. In *Proceedings of the 2021 ACM International Conference on Image and Graphics Processing (ICIGP ’21)* (2021).

[27] Thammarak, K., Kongkla, P., Sirisathitkul, Y. & Intakosum, S. Comparative analysis of tesseract and google cloud vision for Thai vehicle registration certificate. *Int. J. Electr. Comput. Eng. (IJECE)* **12**, 1849–1858. 10.11591/ijece.v12i2.pp1849-1858 (2022).

