# Supplementary material for: Advanced deep learning techniques for automated license plate recognition
Source: Sci Rep. 2025 Nov 21;15:41194. doi: 10.1038/s41598-025-24967-9 (PMC12639091; doi:10.1038/s41598-025-24967-9)
Supplement: Supplementary file 1 — Supplementary Information. [file 41598_2025_24967_MOESM1_ESM.pdf]

Abbreviation	Full Term
AAS	Advanced Augmentation Strategies
AI	Artificial Intelligence
ALPR	Automated License Plate Recognition
ANPR	Automatic Number Plate Recognition
API	Application Programming Interface
CCTV	Closed-Circuit Television
CNN	Convolutional Neural Network
CSP	Cross Stage Partial
CTC	Connectionist Temporal Classification
DCK	Dynamic Convolutional Kernels
EAST	Efficient and Accurate Scene Text
F1-Score	Harmonic Mean of Precision and Recall
FPS	Frames Per Second
GET	Hypertext Transfer Protocol GET Method
GPU	Graphics Processing Unit
IoU	Intersection over Union
JSON	JavaScript Object Notation
LPR	License Plate Recognition
LSTM	Long Short-Term Memory
ML	Machine Learning
P/R	Precision/Recall
PAN	Path Aggregation Network
POST	Hypertext Transfer Protocol POST Method
R-CNN	Region-based Convolutional Neural Network
SC	Special Collection
SPP	Spatial Pyramid Pooling
SSD	Single Shot Multibox Detector
YOLO	You Only Look Once
mAP	Mean Average Precision

Supplementary Table 1. List of commonly used abbreviations in the study.
